# Lactate and pH as Independent Biomarkers for Prognosticating Meaningful Post-out-of-Hospital Cardiac Arrest Outcomes: A Systematic Review and Meta-Analysis

**DOI:** 10.3390/jcm14072244

**Published:** 2025-03-25

**Authors:** Nishil T. Patel, Casey T. Carr, Charlotte M. Hopson, Charles W. Hwang

**Affiliations:** 1 Department of Emergency Medicine, University of Florida, Gainesville, FL 32610, USA; npatel2@anest.ufl.edu (N.T.P.); charlotte.hopson@ufl.edu (C.M.H.); 2 Department of Anesthesiology, Division of Critical Care Medicine, University of Florida, Gainesville, FL 32610, USA; 3 University of Florida College of Medicine, University of Florida, Gainesville, FL 32610, USA; caseycar@ufl.edu; 4 Department of Emergency Medicine, University of Florida, 655 W 8th St, Jacksonville, FL 32209, USA

**Keywords:** out-of-hospital cardiac arrest, lactic acid, hydrogen-ion concentration, blood gas, return of spontaneous circulation

## Abstract

**Background/Objectives**: To systematically review the literature and to characterize the utility of lactate and pH for predicting survival and long-term neurological outcomes after out-of-hospital cardiac arrest (OHCA). **Methods**: PRISMA guidelines were followed. PubMed, Embase, Web of Science, Cochrane Central, and Academic Search Premier were searched for relevant studies. The population included adults with OHCA. Studies with majority in-hospital cardiac arrest (>50%) and studies predicting return of spontaneous circulation (ROSC) were excluded. Pairs of investigators reviewed the studies for relevance. Data were extracted and risk of bias was assessed using the Newcastle–Ottawa Scale. Meta-analyses were performed to characterize the relationship between lactate and pH with survival and neurological outcomes. **Results**: We included 21,120 patients over 49 studies. Most studies (78%) included OHCA only. Mean lactate of 7.24 (95%CI:6.05–8.44) was associated with favorable survival (*n* = 9155; 21 studies), while mean lactate of 7.15 (95%CI:6.37–7.93) was associated with favorable neurological outcome (*n* = 7534; 21 studies). Mean pH of 7.22 (95%CI:7.10–7.33) was associated with favorable survival (*n* = 4077; 7 studies), while a mean pH of 7.22 (95%CI:7.17–7.27) was associated with favorable neurological outcome (*n* = 6701; 13 studies). Poor outcomes were associated with lower pH and higher lactate values. Risk of bias was generally low to medium, while heterogeneity was high. **Conclusions**: A direct correlation exists between pH with survival and neurological outcome; the likelihood of favorable outcomes increases as pH increases. Conversely, an inverse relationship exists between lactate with survival and neurological outcome; higher lactate is associated with poorer outcomes. For lactate, the threshold for survival was more lenient than for favorable neurological outcome.

## 1. Introduction

The annual incidence of out-of-hospital cardiac arrest (OHCA) in the United States is 356,000 per year [1,2]. Survival is poor, and less than 10% of emergency medical services-(EMS)-treated nontraumatic OHCA patients survive to hospital discharge [2,3].

Although return of spontaneous circulation (ROSC) is a crucial step in OHCA survival, ROSC is merely the first step in survival and does not necessarily confer other patient-centered outcomes, such as hospital discharge or favorable neurological recovery. Despite ROSC, patients may experience poor neurological recovery, severe neurological or functional deficits, loss of quality of life, delayed withdrawal of care, and significant psychosocial strain on family members [4,5,6,7,8,9].

Post-cardiac arrest care accounts for USD 5.61 billion annually in the United States. Patients who do not survive to hospital discharge account for nearly 58% of this expenditure [10]. Therefore, significant research has been directed towards identifying specific patient cohorts that will benefit from post-cardiac arrest interventions, such as targeted temperature management [11,12], early coronary angiography [13,14,15,16], and extracorporeal cardiopulmonary resuscitation (ECPR) [17].

Identifying specific variables and thresholds associated with favorable versus unfavorable survival and neurological outcomes is essential in guiding clinicians in medical decision-making and meaningful discussions with families regarding evidence-based expectations.

Lactate and pH are two laboratory markers which are readily available in most hospital contexts and are associated with relative hypoxia time. Prior literature has shown a correlation with these markers and survival [18,19,20,21]. The goal of this systematic review and meta-analysis was to identify specific lactate and pH thresholds beyond which meaningful outcomes and survival may become statistically improbable.

## 2. Materials and Methods

### 2.1. Protocol and Registration

This systematic review and meta-analysis followed the Preferred Reporting Items for Systematic Reviews and Meta-Analyses (PRISMA) guidelines [22]. The PRISMA checklist is presented in the Supplemental Content. The research protocol is provided in the Supplemental Content and was prospectively registered at the International Prospective Register of Systematic Reviews (PROSPERO no. CRD42024563136).

### 2.2. Eligibility Criteria and Outcomes

We used the PICO framework (Population, Intervention, Comparison, Outcome) to frame the study question: in adults (≥18 years) with ROSC after nontraumatic OHCA (P), does higher serum pH or lower serum lactate (I), as compared to lower pH or higher lactate (C), have any impact on long-term patient-centered outcomes (O)?

The outcomes included survival and neurological outcomes at and after hospital discharge. Favorable neurological outcome was defined as a Cerebral Performance Category (CPC) score of 1–2, modified Rankin Scale (mRS) of 0–3, or Glasgow outcome scale (GOS) of 4–5. Poor neurological outcome was defined as a CPC of 3–5, mRS of 4–6, or GOS of 1–3. Included time intervals ranged from 24 h to 1 year after OHCA.

Randomized controlled trials, non-randomized controlled trials, and observational studies with a comparison group were included. Animal studies, reviews, abstracts, editorials, comments, and letters to the editor were excluded. Studies using intra-arrest laboratory values to predict ROSC were excluded. Studies evaluating serum pCO_2_ or analyzing labs prior to ECPR initiation were excluded. Mixed studies containing both OHCA and in-hospital cardiac arrest (IHCA) patients were excluded if IHCA patients represented more than 50% of the study population or if the IHCA:OHCA ratio was not reported. Studies primarily focusing on pediatric patients were excluded. There were no limitations on publication period or manuscript language (provided an English abstract existed).

### 2.3. Information Sources and Search Strategy

The search terms and strategy were developed in collaboration with a research librarian specializing in systematic reviews. We searched PubMed, Embase, Web of Science, Cochrane Central, and Academic Search Premier databases on 7 December 2022. We performed an updated search using the same search strategy and databases on 15 August 2024 to include papers from December 2022 to August 2024. The search strategy is included in the Supplemental Content.

### 2.4. Study Selection

Using pre-determined screening criteria, pairs of reviewers independently screened all titles and abstracts retrieved by the query. Kappa statistics were calculated to determine inter-rater agreement. Any discrepancies regarding inclusion and exclusion of screened articles were resolved by discussion between the reviewer pair, with a third reviewer adjudicating unresolved discrepancies. Articles selected for full-text appraisal were assessed by a pair of reviewers and yielded a final selection of articles for data extraction. Disagreements regarding article eligibility were resolved by discussion between the reviewer pair.

### 2.5. Data Collection

Using a predefined data extraction tool (provided in Supplemental Content), data were independently extracted from each included article by a pair of reviewers. Briefly, extracted data included study design, inclusion and exclusion criteria, population statistics (e.g., age, sex, sample size), and exposure (i.e., pH or lactate) with its respective outcomes (i.e., survival or neurological outcome) and statistics (e.g., mean, median, odds ratios, etc.). Missing data were calculated from provided data if possible. Discrepancies in extracted data were resolved by discussion and consensus decision.

### 2.6. Risk of Bias for Individual Studies

Given the nature of the PICO, it was projected that most included articles would be nonrandomized studies. The Newcastle–Ottawa scale (NOS) is an instrument developed to systematically assess quality for nonrandomized studies in a systematic review [23]. Therefore, for each included article, two authors independently evaluated the risk of bias using the NOS, and disagreements regarding quality scoring were resolved by discussion.

### 2.7. Data Synthesis and Analysis

Using the extracted data, the main outcome measures of interest were dichotomized into favorable versus poor (i.e., favorable survival, poor survival, favorable neurological outcome, poor neurological outcome). Means and standard deviations, if available, were extracted and used for effect size. If the mean was available but not the standard deviation, the standard deviation was estimated using the reported *p*-value or interquartile range. For studies only reporting medians and interquartile ranges, methods from Luo et al. [24] were used to estimate the mean, and methods from Wan et al. [25] were used to estimate the standard deviation.

#### 2.7.1. Mean Analysis

A meta-analysis of means (pH and lactate) by outcome type was performed using the meta package in R by pooling the raw means and standard deviations using the restricted maximum-likelihood estimator and the inverse variance method [26,27]. Random-effects models for the comparative outcome groups were run, and the Hartung–Knapp adjustment was applied.

Some degree of heterogeneity was expected as the studies analyzed different outcome measures at varying time intervals. Therefore, a “full” meta-analysis including all studies was performed. A final model excluded outliers [28]. Forest plots for the final models were created using the metafor package [29].

A meta-analysis of standardized mean differences (pH and lactate) between outcomes was performed in a similar fashion.

#### 2.7.2. Odds Ratio Analysis

Using studies that reported pH and lactate odds ratios for outcomes, a meta-analysis was performed for pH and lactate odds ratios. Extracted odds ratios and confidence intervals were log-transformed and pooled using the inverse variance method in random-effects models. The Paule–Mandel estimator was used to calculate τ^2^.

## 3. Results

### 3.1. Identified Studies

**Figure 1:**
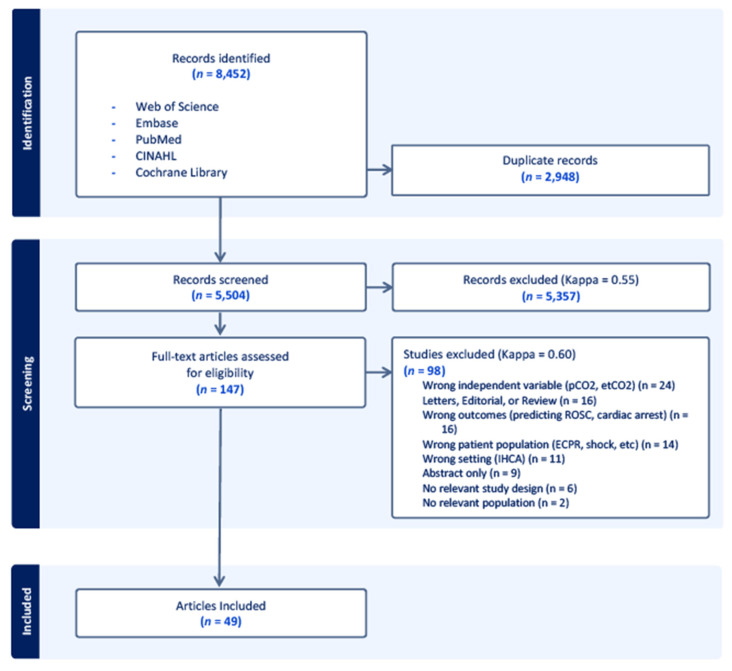
PRISMA diagram demonstrating the selection of articles during the review process. Out of 5504 screened articles, 147 articles underwent full-text analysis to determine eligibility, and 49 articles were ultimately included for data extraction. Of the included studies, 36 were retrospective cohort studies, 11 were prospective observational cohort studies, one was a combined retrospective and prospective cohort study, and one study was randomized.

The search query identified 5504 unique records, of which 5357 records were excluded after review of the titles and abstracts. The Kappa for the initial screening was 0.55. Of the 147 full-text article reviews, 98 were excluded (Kappa = 0.60) for the reasons listed in Figure 1, leaving a total of 49 articles [9,18,19,20,21,30,31,32,33,34,35,36,37,38,39,40,41,42,43,44,45,46,47,48,49,50,51,52,53,54,55,56,57,58,59,60,61,62,63,64,65,66,67,68,69,70,71,72,73]. Several studies passed initial screening, but were excluded during the full-text review because they consisted of a majority IHCA (i.e., >50%) or they included both IHCA and OHCA but did not specify a percentage of each [74,75,76].

### 3.2. Overview of Included Studies

Of the 49 included articles, 36 articles were retrospective observational cohort studies, 11 were prospective observational cohort studies, one was a prospective randomized controlled trial, and one was a combined retrospective and prospective observational cohort study. Only one study was randomized [71]. The studies included between 32 and 4189 patients, and nine studies included more than 500 patients. Altogether, the studies included 21,120 patients. Studies were conducted in Asia (n = 22), Europe (n = 14), North America (n = 11), Australia (n = 1), and multicontinental (n = 1). All studies were described as including only adult patients. Two studies included ages 16 and above [39,58]; due to the apparent very small number of patients in these studies under the age of 18 and the difficulty in separating these few patients out, the decision was made to include the studies. A brief overview of these studies is provided in Table 1 and detailed information including the results is provided in Appendix A and the Supplemental Content.

#### 3.2.1. Lactate and Survival Analysis

**Figure 2:**
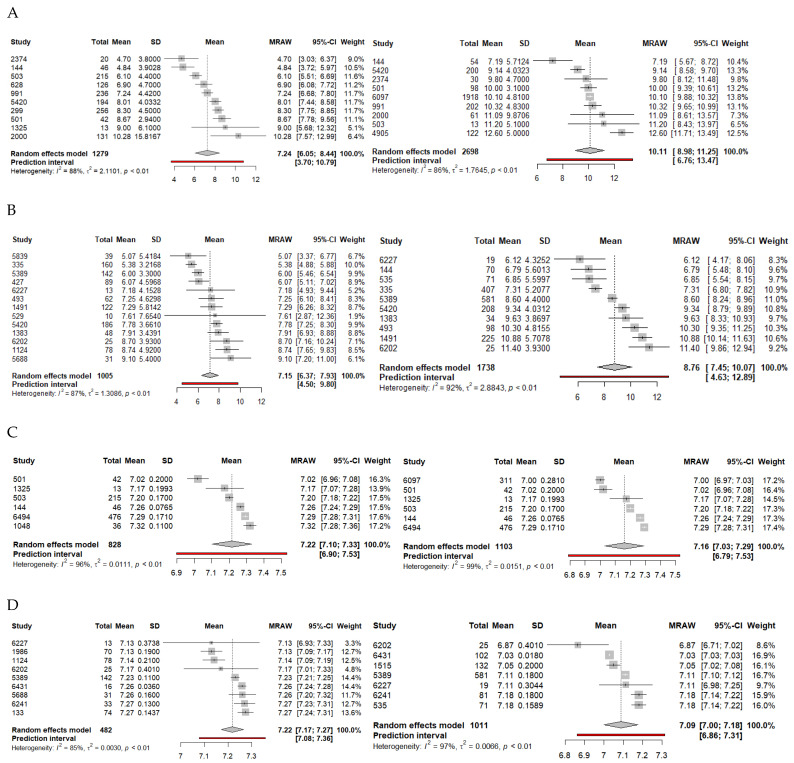
Forest plots for survival (**A**) and neurological outcome (**B**) relative to lactate after outliers are removed. Forest plots for survival (**C**) and neurological outcome (**D**) relative to pH after outliers are removed. Favorable outcomes are on the left, and unfavorable outcomes are on the right. Abbreviations: CI, confidence interval; MRAW, raw mean; SD, standard deviation.

Twenty-one articles compared lactate levels with survival outcome. Sixteen studies were retrospective cohort studies, while five were prospective observational studies for a total of 9155 patients (favorable survival, 3563; poor survival, 5592). Outcome duration ranged from hospital admission to 6-month survival. Means and standard deviations were deduced for 11 studies. In the final model after outliers were removed (n = 3977), a mean lactate of 10.11 (95%CI: 8.98–11.25, *p* < 0.0001) was associated with poor survival, while a mean lactate of 7.24 (95%CI: 6.05–8.44, *p* < 0.0001) was associated with favorable survival (Table 2, Figure 2A).

The random effects model provided a mean-difference effect size of 2.21 (95%CI: 1.76–2.65, *p* < 0.0001) after outliers were removed, indicating a lactate difference of nearly 2 between patients with favorable versus poor survival outcomes.

#### 3.2.2. Lactate and Neurological Outcome Analysis

Twenty-one articles compared lactate levels with neurological outcome. Fifteen studies were retrospective studies, five were prospective observational studies, and one was a prospective randomized clinical trial for a total of 7534 patients (favorable neurological outcome, 1730; poor neurological outcome, 5804). Outcome duration ranged from hospital discharge to one-year neurological outcome. Means and standard deviations were deduced for 15 studies. In the final model after outliers were removed (n = 2743), a mean lactate of 8.76 (95%CI: 7.45–10.07, *p* < 0.0001) was associated with poor neurological outcome, while a mean lactate of 7.15 (95%CI: 6.37–7.93, *p* < 0.0001) was associated with favorable neurological outcome (Table 2, Figure 2B).

The random effects model provided a mean-difference effect size of 2.28 (95%CI: 1.91–2.64, *p* < 0.0001) after outliers were removed, indicating a lactate difference of nearly 2 between patients with favorable versus poor neurological outcomes.

#### 3.2.3. pH and Survival Analysis

Seven articles compared pH levels with survival outcome. Six studies were retrospective studies, while one was a prospective observational study for a total of 4077 patients (favorable survival, 1139; poor survival, 2305). Outcome duration ranged from hospital admission to 28-day survival. Means and standard deviations were deduced for four studies. In the final model (n = 1931), a mean pH of 7.16 (95%CI: 7.03–7.29, *p* < 0.0001) was associated with poor neurological outcome, while a mean pH of 7.22 (95%CI: 7.10–7.33, *p* < 0.0001) was associated with favorable survival (Table 2, Figure 2C).

The random effects model found a mean-difference effect size of −0.09 (95%CI: −0.16, −0.02; *p* = 0.18).

#### 3.2.4. pH and Neurological Outcome Analysis

Thirteen articles compared pH levels with neurological outcome. Nine studies were retrospective studies, while four were prospective observational studies for a total of 6701 patients (favorable neurological outcome, 781; poor neurological outcome, 5920). Outcome duration ranged from hospital discharge to 6-month neurological outcome. Means and standard deviations were deduced for seven studies. In the final model after outliers were removed (n = 1493), a mean pH of 7.09 (95%CI: 7.00–7.18, *p* < 0.0001) was associated with poor neurological outcome, while a mean pH of 7.22 (95%CI: 7.17–7.27, *p* < 0.001) was associated with favorable neurological outcome (Table 2, Figure 2D).

The random effects model found a mean-difference effect size of −0.13 (95%CI: −0.16, −0.10; *p* < 0.0001) after outliers were removed, indicating a pH difference of nearly −0.13 between patients with favorable versus poor neurological outcomes.

#### 3.2.5. Risk of Bias

Applying the Newcastle–Ottawa Scale found that out of 49 studies, 8 were found to have a medium risk of bias (7%) [36,37,39,40,42,48,53,57]. The remaining were rated as having a low risk of bias. Details regarding bias assessments are provided in Table 3 and the Supplemental Content.

Three studies [9,30,57] did not present means, medians, or odds ratios relative to the measured outcomes. When considering each prognostic marker (i.e., lactate or pH) and their respective outcomes, the five medium risk studies were distributed as follows: lactate and neurological outcome (one included in final mean model, one excluded as an outlier, one included in final odds ratio model), lactate and survival (one included in final mean model, one excluded as an outlier, one included in final odds ratio model), pH and neurological outcome (one excluded as an outlier), pH and survival (zero included, zero excluded).

## 4. Discussion

Favorable outcomes after OHCA are possible; therefore, it is important to ascertain the potential for recovery to make educated, evidence-based decisions and to guide family discussions. Our meta-analysis found that the likelihood of survival and favorable neurological outcome increases as pH increases. Conversely, an increase in lactate is associated with poorer outcomes.

Another goal of the meta-analysis was to identify specific lactate and pH values beyond which meaningful recovery would be statistically improbable. We found that the pooled mean lactate associated with poor survival was 10.11 (95%CI: 8.98–11.25, *p* < 0.0001) while the mean lactate associated with favorable survival was 7.24 (95%CI: 6.05–8.44, *p* < 0.0001). The mean lactate associated with poor neurological outcome was 8.76 (95%CI: 7.45–10.07, *p* < 0.0001) while the mean lactate associated with favorable neurological outcome was 7.15 (95%CI: 6.37–7.93, *p* < 0.0001). This would suggest that a lactate of 11.25 is specific for poor survival while 10.07 is specific for poor neurological outcome. A lactate of 8.44 is sensitive for favorable survival, and 7.93 is sensitive for favorable neurological outcome.

Similarly, a pH of 7.03 is specific for poor survival while 7.00 is specific for poor neurological outcome. A pH of 7.10 is sensitive for favorable survival, and 7.17 is sensitive for favorable neurological outcome.

Put differently, a lactate greater than 11.25 or a pH less than 7.03 is highly suggestive of poor long-term survival, and a lactate greater than 10.07 or a pH less than 7.00 is highly suggestive of poor neurological outcome.

For lactate, the thresholds for favorable survival were more lenient than the thresholds for favorable neurological outcome (e.g., lactate of 7.24 vs. 7.15, respectively). The survival–neurological outcome relationship is consistent with Cardiac Arrest Registry to Enhance Survival (CARES) data [2]. The survival–neurological outcome relationship is also consistent with data derived from studies evaluating post-hypoxic organ injury in potential organ donors [77]. A patient may survive a hypoxic insult but have poor neurologic prognosis.

Careful analysis of our data shows that heterogeneity may provide a strong foundation for generalizability. Our *I^2^* values ranged from 71 to 99%, which represents moderately high heterogeneity and classically raises practical questions regarding the effect size. However, pragmatic studies accept all-comers from a particular group of interest, which is found in clinical practice, and are therefore more applicable to broader populations and face fewer issues with external validation.

Sampling differences could explain the variation in our lactate data. Despite the heterogeneity, the τ^2^ is very low, suggesting that the variation is low despite the wide range of effect sizes between included studies. Furthermore, after removing outliers, the calculated mean demonstrated minimal change in nearly every case, further reinforcing the notion that variability is low. Therefore, the calculated pH and lactate averages are likely comparable to the actual means and can be useful for prognostication.

Similar to our data, Seeger et al. [53] found that high lactate and low pH on admission were associated with death or severe hypoxic brain injury within 30 days after OHCA. Takaki et al. [58] also found significantly higher pH levels in patients with favorable 6-month neurological recovery. In contrast, a 2018 study by Dadeh et al. [66] showed no correlation between initial serum lactate with survival to hospital discharge. This is likely due to the limits of a single-center study with only 207 patients, which limits its generalizability, power, and inability to control confounding factors.

The 49 included studies were dichotomized into two groups using the Newcastle–Ottawa score, ≥7 (low risk of bias) or <7 (medium to high risk of bias). Forty-one out of forty-nine studies (83.7%) received a score ≥7 by independent scoring and majority agreement between primary authors. The results suggest there is low risk of bias within the systematic review and the quality of the original articles is high. Strict control of inclusion criteria ensured the systematic review closely resembles the intended study population. Moreover, outcomes were robustly documented given electronic medical record (EMR) utilization and record linkage. Comparison between cohorts is limited because patient-centered outcomes are dependent on multiple pre-existing patient factors such as age, gender, and comorbidities that are impossible to control; this was the principal contributor of bias.

Prospective validation of our findings is required. This systematic review and meta-analysis demonstrates that a correlation exists between lactate and pH with long-term outcomes. Future studies will need to be performed to validate our findings and to develop a clinical decision tool.

### Limitations

Limitations include the retrospective nature and the natural heterogeneity of the meta-analysis, such as varying demographics, comorbidities, and inclusion criteria. A few other limitations warrant discussion. While most included studies exclusively enrolled patients who suffered OHCA, a few studies also included a small portion of IHCA. None of these had >50% IHCA, and in our sample, IHCA represented only a small minority of patients.

One major area of heterogeneity was the follow-up length. Most studies had at least 30-day survival listed as a major outcome, with a few reporting data through 90 days or longer. A smaller number had less common milestones such as 14, 45, or 60-day survival. In total, the resulting 30-day survival marker utilized in our analysis was preserved.

Another source of heterogeneity was time from ROSC to collection of initial samples. “Post-ROSC” times have variable definitions, from immediately after ED arrival to several hours after ROSC. This does not consider pre-hospital down-time, nor laboratory sampling errors, sampling variations between equipment, and variations in laboratory analyses. The above factors could explain the wide range in our data for lactate (~3.8–~12.8). pH, on the other hand, had a tighter range, which can be explained by the body’s homeostasis mechanisms and physiological buffering systems. Nevertheless, many studies included lactate and blood gas measurements within 24 h of ROSC, a critical portion of time in the post-arrest period. The consistent nature of survival having a more lenient threshold than neurological outcome longitudinally across all examined studies speaks to the internal validity of our results.

Additionally, our results show that removing outliers had minimal impact on our results despite the presence of heterogeneity. An interpretation of this could be that many of the unknown confounding factors are self-controlled, and the true population means for pH and lactate lie within our predicted range.

## 5. Conclusions

Out-of-hospital cardiac arrest accounts for significant morbidity, mortality, and healthcare spending in the United States and around the world. Initial post-ROSC serum lactate and pH values can be powerful prognostic indicators for meaningful recovery as defined by the patient’s wishes and family. Most importantly, these tools can help clinicians educate families and adjudicate resources with an informed approach.

## Figures and Tables

**Table 1:** Overview of all included studies.

				Exposures and Outcomes				Studies Reporting Results Based on Cutoffs					Results Based on Population-Level Statistics		
Study	Years of Inclusion	OHCA vs. IHCA	Study Design	Exposure (pH or Lactate)	Outcome	Sample Size	Data Type	Cutoff	Exposure < Cutoff		Exposure > Cutoff		Median (IQR) or Mean (SD) *	Other Results (e.g., OR, HR, AUC, etc.)	*p*-Value
							(n, median [IQR], mean [SD], OR)								
									n	%	n	%			
Al Assil et al., 2021 [30]	2011–2015	OHCA	Retrospective cohort	pH	mRS 0–3 at discharge	1345	n	7.08	131	9.7%	1214	90.3%			<0.001
				pH	mRS 4–6 at discharge	2844	n	7.08	880	30.9%	1964	69.1%			<0.001
				pH	mRS 0–3 at discharge	1345	n	7.21	397	29.5%	948	70.5%			<0.001
				pH	mRS 4–6 at discharge	2844	n	7.21	1617	56.9%	1227	43.1%			<0.001
				pH	mRS 0–3 at discharge	1345	n	7.3	892	66.3%	453	33.7%			<0.001
				pH	mRS 4–6 at discharge	2844	n	7.3	2240	78.8%	604	21.2%			<0.001
Carr et al., 2020 [18]	2016–2018	OHCA	Retrospective cohort	pH	Survival to discharge	13	n	7.2	6	46.2%	7	53.8%			<0.001
				pH	Died prior to discharge	66	n	7.2	61	92.4%	5	7.6%			<0.001
				pH	Survival to discharge	13	Median (IQR)						7.21 (7.04–7.28)		<0.001
				pH	Died prior to discharge	66	Median (IQR)						6.90 (6.82–7.02)		<0.001
				Lactate	Survival to discharge	13	Mean (SD)						9 (6.1) *		0.03
				Lactate	Died prior to discharge	66	Mean (SD)						12.4 (4.7) *		0.03
				pH	Survival to discharge	79	OR	7.2						0.08 (0.02–0.32)	<0.001
Chen et al., 2024 [73]	2015–2023	OHCA	Retrospective cohort	Lactate	CPC 1–2 at 30 days	87	Mean (SD)						10.3 (5.4) *		<0.001
				Lactate	CPC 3–5 at 30 days	132	Mean (SD)						13.0 (6.0) *		<0.001
				pH	CPC 1–2 at 30 days	87	Mean (SD)						7.13 (0.18) *		0.01
				pH	CPC 3–5 at 30 days	132	Mean (SD)						7.05 (0.20) *		0.01
Choi et al., 2024 [72]	2009–2022	OHCA	Retrospective cohort	Lactate	CPC 1–2 at 6 months	122	Median (IQR)						6.85 (3.60–11.35)		<0.001
				Lactate	CPC 3–5 at 6 months	225	Median (IQR)						10.90 (7.05–14.70)		<0.001
Cocchi et al., 2020 [19]	2008–2016	OHCA	Prospective observational cohort	Lactate	Survival to discharge	96	n	5	54	56.3%	42	43.8%			<0.001
				Lactate	Died prior to discharge	152	n	5	45	29.6%	107	70.4%			<0.001
				Lactate	Survival to discharge	96	n	10	88	91.7%	8	8.3%			<0.001
				Lactate	Died prior to discharge	132	n	10	93	70.5%	39	29.5%			<0.001
				Lactate	Survival to discharge	97	Median (IQR)						4.1 (2.8–6.9)		<0.001
				Lactate	Died prior to discharge	152	Median (IQR)						6.8 (4.2–10.1)		<0.001
				Lactate	Died prior to discharge	99	OR	<5						1 (reference range)	
				Lactate	Died prior to discharge	103	OR	5–10						2.33 (1.32–4.1)	0.004
				Lactate	Died prior to discharge	47	OR	>10						5.85 (2.48–13.8	<0.001
Cocchi et al., 2011 [31]	2006–2008 (A) and 2006–2007 (B)	OHCA	Retrospective cohort	Lactate	Survival to discharge	37	n	5	22	59.5%	15	40.5%			<0.0001
				Lactate	Died prior to discharge	91	n	5	14	15.4%	77	84.6%			<0.0001
				Lactate	Survival to discharge	37	n	10	32	86.5%	5	13.5%			<0.0001
				Lactate	Died prior to discharge	91	n	10	34	37.4%	57	62.6%			<0.0001
				Lactate	Died prior to discharge	36	OR	<5						1 (reference range)	
				Lactate	Died prior to discharge	30	OR	5–10						3.1 (1.1–8.7)	0.03
				Lactate	Died prior to discharge	62	OR	>10						17.9 (5.8–55.6)	<0.0001
Dadeh et al., 2018 [66]	2015–2017	OHCA	Retrospective cohort	Lactate	Survival to discharge	85	Mean (SD)						12 (4.8) *		0.381
				Lactate	Died prior to discharge	122	Mean (SD)						12.6 (5) *		0.381
Dell’Anna et al., 2017 [32]	2009–2013	Combined (58% OHCA)	Prospective observational cohort	Lactate	CPC 1–2 at three months	74	Median (IQR)						2.5 (1.5–5.5)		<0.001
				Lactate	CPC 3–5 at three months	162	Median (IQR)						5.3 (2.9–9.0)		<0.001
				pH	CPC 1–2 at three months	74	Median (IQR)						7.27 (7.18–7.37)		0.09
				pH	CPC 3–5 at three months	162	Median (IQR)						7.25 (7.15–7.34)		0.09
Donnino et al., 2014 [20]	2011–2012	OHCA	Retrospective cohort	pH	Survival to discharge	46	Median (IQR)						7.3 (7.2–7.3)		0.02
				pH	Died prior to discharge	54	Median (IQR)						7.2 (7.1–7.3)		0.02
				Lactate	Survival to discharge	46	Median (IQR)						4.1 (2.6–7.7)		0.004
				Lactate	Died prior to discharge	54	Median (IQR)						7.3 (3.4–10.9)		0.004
				Lactate	mRS 0–3 at discharge	30	Median (IQR)						3.9 (2.7–6.1)		0.009
				Lactate	mRS 4–6 at discharge	70	Median (IQR)						7.0 (3.0–10.4)		0.009
				Lactate	AUC for predicting mortality		AUC							0.67	
				Lactate	AUC for predicting mRS 4–6 at discharge		AUC							0.67	
During et al., 2018 [33]	2010–2013	OHCA	Retrospective cohort	Lactate	Survival to 30 days	486	n	6	297	61.1%	189	38.9%			<0.001
				Lactate	Died prior to 30 days	391	n	6	147	37.6%	244	62.4%			<0.001
				Lactate	CPC 1–2 at 180 days	408	n	6	250	61.3%	158	38.7%			<0.001
				Lactate	CPC 3–5 at 180 days	469	n	6	194	41.4%	275	58.6%			<0.001
				Lactate	Died prior to 30 days	391	Median (IQR)						7.30 (4.5–10.7)		
				Lactate	Survival to 30 days	486	Median (IQR)						4.65 (2.4–8)		
Dusik et al., 2023 [71]	2013–2020	OHCA	Prospective randomized clinical trial	Lactate	CPC 1–2 from 30–180 days	48	Median (IQR)						7.8 (5.7–10.2)		0.055
				Lactate	CPC 3–5 from 30–180 days	34	Median (IQR)						9.7 (7.1–12.1)		0.055
Freire-Jorge et al., 2021 [34]	2006–2016	OHCA	Retrospective cohort	Lactate	Survival to discharge	82	Mean (95% CI)						12.4 (11.4–12.9)		0.951
				Lactate	Died prior to discharge	73	Mean (95% CI)						12.2 (11.2–13.1)		0.951
Han et al., 2019 [35]	2006–2017	OHCA	Prospective observational cohort	Lactate	Survival to discharge	145	Mean (SD)						9.2 (4.0) *		<0.001
				Lactate	Died prior to discharge	190	Mean (SD)						11.9 (5.1) *		<0.001
Hope Kilgannon et al., 2019 [36]	2013–2017	Combined (77% OHCA)	Prospective observational cohort	pH	mRS 0–3 at discharge	85	Mean (95% CI)						7.28 (7.22–7.36) *		0.007
				pH	mRS 4–6 at discharge	195	Mean (95% CI)						7.26 (7.17–7.33) *		0.007
Imamura et al., 2023 [70]	2015–2022	OHCA	Retrospective cohort	pH	mRS 0–3 at 30 days	78	Mean (SD)						7.14 (0.21) *		<0.001
				pH	mRS 4–6 at 30 days	116	Mean (SD)						6.95 (0.17) *		<0.001
				lactate	mRS 0–3 at 30 days	78	Mean (SD)						8.74 (4.92) *		<0.001
				lactate	mRS 4–6 at 30 days	116	Mean (SD)						12.57 (4.85) *		<0.001
Kandilcik et al, 2024 [69]	2017–2020	Combined (70% OHCA)	Retrospective cohort	pH	Survival to hospital discharge	36	Mean (SD)						7.32 (0.11) *		0.028
				pH	Died prior to hospital discharge	115	Mean (SD)						7.26 (0.15) *		0.028
				Lactate	Survival to hospital discharge	36	Median (Min-Max)						1.9 (0.6–16)		<0.001
				Lactate	Died prior to hospital discharge	115	Median (Min-Max)						5.1 (0.7–20)		<0.001
Kei et al., 2017 [37]	2012–2013	OHCA	Prospective observational cohort	Lactate	Survival to 30 days	256	Mean (SD)						8.3 (4.5) *		<0.001
				Lactate	Died prior to 30 days	287	Mean (SD)						11.3 (5.3) *		<0.001
Kiehl et al., 2019 [38]	2008–2014 (UVA) and 2012 to 2017 (CCF)	OHCA	Prospective observational cohort	Lactate	CPC 1–2 at discharge	142	Mean (SD)						6.0 (3.3) *		<0.001
				Lactate	CPC 3–5 at discharge	581	Mean (SD)						8.6 (4.4) *		<0.001
				pH	CPC 1–2 at discharge	142	Mean (SD)						7.23 (0.11) *		<0.001
				pH	CPC 3–5 at discharge	581	Mean (SD)						7.11 (0.18) *		<0.001
Kim et al., 2023 [68]	2016–2020	OHCA	Retrospective cohort	Lactate	Hospital discharge	236	Mean (SD)						7.24 (4.42) *		<0.001
				Lactate	Hospital discharge	202	Mean (SD)						10.32 (4.83) *		<0.001
Kim et al., 2017 [39]	2012–2015	Combined (81% OHCA)	Retrospective cohort	Lactate	CPC 3–5 at discharge	282	OR (95% CI)							1.049 (0.962–1.143)	
				Lactate	Died prior to discharge	282	OR (95% CI)							1.063 (0.981–1.152)	
Kliegel et al., 2004 [40]	1991–2001	OHCA	Retrospective cohort	Lactate	Survival to 6 months	194	Median (IQR)						7.8 (5.4–10.8)		<0.01
				Lactate	Died prior to 6 months	200	Median (IQR)						9 (6.5–11.9)		<0.01
				Lactate	CPC 1–2 at 6 months	186	Median (IQR)						7.6 (5.4–10.3)		<0.001
				Lactate	CPC 3–5 at 6 months	208	Median (IQR)						9.2 (6.7–12.1)		<0.001
Laurikkala et al., 2019 [41]	2010–2011	OHCA	Prospective observational cohort	Lactate	CPC 1–2 at 1 year	185	Median (IQR)						3.06 (2.68–3.44)		<0.001
				Lactate	CPC 3–5 at 1 year	273	Median (IQR)						4.76 (4.29–5.23)		<0.001
Lee et al., 2015 [42]	2007–2012	OHCA	Retrospective cohort	Lactate	Survival to discharge	254	Mean (SD)						9.55 (4.33) *		Authors state “statistically significant, no *p* value given
				Lactate	Died prior to discharge	289	Mean (SD)						11.36 (4.58) *		Authors state “statistically significant, no *p* value given
				Lactate	CPC 1–2 at discharge	96	Mean (SD)						8.99 (4.17) *		Not specified
				Lactate	CPC 3–5 at discharge	347	Mean (SD)						10.70 (4.55) *		Not specified
Lin et al., 2021 [43]	2012–2018	OHCA	Retrospective cohort	pH	CPC 1–2 at discharge	70	Mean (SD)						7.13 (0.19) *		<0.001
				pH	CPC 3–5 at discharge	1964	Mean (SD)						6.98 (0.17) *		<0.001
				pH	CPC 3–5 at discharge	1964	OR (univariate)							0.02 (0.01–0.05)	<0.001
				pH	CPC 3–5 at discharge	1964	OR (multivariate)							0.03 (0.01–0.13)	<0.001
				pH	CPC 1–2 at discharge	2034	AUC							0.7316	
Lonsain et al., 2021 [44]	2012–2019	OHCA	Retrospective cohort	Lactate	Survival to 48 h	152	Median (IQR)						8.2 (0.9–22)		no *p* value reported
				Lactate	Died prior to 48 h	24	Median (IQR)						11.3 (3.7–29.0)		no *p* value reported
				Lactate	Survival to 72 h	131	Median (IQR)						7.5 (0.9–22.0)		no *p* value reported
				Lactate	Died prior to 72 h	61	Median (IQR)						10.1 (5.0–18.0)		no *p* value reported
Marinšek et al., 2020 [9]	2014–2016	OHCA	Retrospective cohort	Lactate	CPC 1–2 at 72 h	41	n	6	38	92.7%	3	7.3%			<0.001
				Lactate	CPC 3–5 at 72 h	69	n	6	39	56.5%	30	43.5%			<0.001
Momiyama et al., 2017 [21]	2010–2013	OHCA	Retrospective cohort	Lactate	CPC 1–2 at discharge	31	Mean (SD)						9.1 (5.4) *		“not significant”
				Lactate	CPC 3–5 at discharge	341	Mean (SD)						10.7 (4.6) *		“not significant”
				pH	CPC 1–2 at discharge	31	Mean (SD)						7.26 (0.16) *		<0.001
				pH	CPC 3–5 at discharge	341	Mean (SD)						6.93 (0.19) *		<0.001
Orban et al., 2017 [45]	2006–2013	OHCA	Retrospective cohort	Lactate	CPC 1–2 at discharge	89	Median (IQR)						5.4 (3.3–9.4)		< 0.01
				Lactate	CPC 3–5 at discharge	183	Median (IQR)						2.2 (1.5–3.6)		< 0.01
				Lactate	CPC 3–5 at discharge	272	OR (95% CI)	Lactate > 4						7.54 (4.07–14.0)	<0.0001
Park et al., 2019 [46]	2011–2016	OHCA	Retrospective cohort	Lactate	CPC 1–2 at discharge	39	Median (IQR)						3.98 (2.00–9.04)		0.492
				Lactate	CPC 3–5 at discharge	63	Median (IQR)						4.96 (3.20–9.43)		0.492
Peluso et al., 2020 [47]	2009–2017	Combined (65% OHCA)	Retrospective cohort	Lactate	Survival to discharge	147	Median (IQR)						4.3 (3.0–7.1)		<0.05
				Lactate	Died prior to discharge	209	Median (IQR)						7.0 (4.6–9.2)		<0.05
				Lactate	CPC 1–2 at 3 months	126	Median (IQR)						4.2 (2.8–6.4)		<0.05
				Lactate	CPC 3–5 at 3 months	230	Median (IQR)						6.8 (4.6–9.3)		<0.05
				Lactate	CPC 3–5 at 3 months	356	OR							1.16 (1.06–1.29)	0.002
				Lactate	Died prior to discharge	356	OR							1.11 (1.02–1.19)	0.017
Rezar et al., 2021 [48]	2018–2020	Combined (84% OHCA)	Retrospective cohort	Lactate	Died prior to 30 days	106	HR							1.15 (1.07–1.24)	<0.001
				Lactate	Died prior to 30 days	106	AUC							0.75 (0.66–0.83)	0.11
				Lactate	Survival to 30 days	72	n	2.5	46	63.9%	26	36.1%			
				Lactate	Died prior to 30 days	32	n	2.5	5	15.6%	27	84.4%			
Rosenberg et al., 2021 [49]	2014–2018	Combined (76% OHCA)	Retrospective cohort	Lactate	Survival to discharge	20	Mean (SD)						4.7 (3.8)*		<0.01
				Lactate	Died prior to discharge	30	Mean (SD)						9.8 (4.7)*		<0.01
				Lactate	Died prior to discharge	50	OR							1.39 (1.13–1.71)	<0.01
				Lactate	Died prior to discharge	50	aOR							1.56 (1.19–2.05)	<0.01
				Lactate	Survival to discharge	20	n	4.5	12	60.0%	8	40.0%			<0.05
				Lactate	Died prior to discharge	30	n	4.5	4	13.3%	26	86.7%			<0.05
				Lactate	Survival to discharge	20	n	9	17	85.0%	3	15.0%			<0.05
				Lactate	Died prior to discharge	30	n	9	16	53.3%	14	46.7%			<0.05
Ryoo et al., 2020 [50]	2013–2018	OHCA	Retrospective cohort	Lactate	CPC 3–5 at 28 days	98	Median (IQR)						10.3 (7.1–13.5)		<0.05
				Lactate	CPC 1–2 at 28 days	62	Median (IQR)						7.5 (4.1–10.2)		<0.05
Sarıaydın et al., 2017 [51]	2015–2016	OHCA	Prospective observational cohort	Lactate	Survival to 24 h	42	Mean (SD)						8.67 (2.94) *		0.1
				Lactate	Died prior to 24 h	98	Mean (SD)						10 (3.1) *		0.1
				pH	Survival to 24 h	42	Mean (SD)						7.02 (0.2) *		0.7
				pH	Died prior to 24 h	98	Mean (SD)						6.96 (0.17) *		0.7
Sauter et al., 2017 [52]	2012–2015	OHCA	Retrospective cohort	pH	Survival to hospital admission	215	Mean (SD)						7.2 (0.17) *		0.01
				pH	Died prior to hospital admission	13	Mean (SD)						7.01 (0.13) *		0.01
				Lactate	Survival to hospital admission	215	Mean (SD)						6.1 (4.4) *		0.013*
				Lactate	Died prior to hospital admission	13	Mean (SD)						11.2 (5.1) *		0.01
Seeger et al., 2013 [53]	2007–2009 (retrospective) and 2009–2010 (prospective)	Combined (65% OHCA)	Retrospective Cohort and Prospective observational cohort	Lactate	Combination of death or severe hypoxic brain damage within 30 days	206	univariate HR (95CI)	Lactate> 6.94						2.772 (1.953–3.936)	<0.001
				pH	Combination of death or severe hypoxic brain damage within 30 days	206	univariate HR (95CI)	pH < 7.21						2.706 (1.912–3.830)	<0.001
				Lactate	Combination of death or severe hypoxic brain damage within 30 days	206	multivariate HR	Lactate> 6.94						2.026 (1.371–2.994)	<0.001
				pH	Combination of death or severe hypoxic brain damage within 30 days	206	multivariate HR	pH < 7.21						2.027 (1.342–3.060)	0.001
Shin et al., 2017 [54]	2009–2014	OHCA	Retrospective cohort	pH	Survival to discharge	311	Mean (IQR)						7.00 (6.93–7.31)		<0.001
				pH	Died prior to discharge	1918	Mean (IQR)						6.96 (6.83–7.20)		<0.001
				pH	CPC 1–2 at 28 days	98	Mean (IQR)						7.11 (7.00–7.26)		<0.001
				pH	CPC 3–5 at 28 days	2131	Mean (IQR)						6.96 (6.84–7.09)		<0.001
				Lactate	Survival to discharge	311	Mean (IQR)						9.5 (6.9–11.7)		0.006
				Lactate	Died prior to discharge	1918	Mean (IQR)						10.1 (7.1–13.6)		0.006
				Lactate	CPC 1–2 at 28 days	98	Mean (IQR)						8.7 (6.8–10.8)		0.011
				Lactate	CPC 3–5 at 28 days	2131	Mean (IQR)						10.1 (7.1–13.4)		0.011
Shinozaki et al., 2011 [55]	2007–2009	OHCA	Prospective observational cohort	Lactate	CPC 1–2 at 6 months	10	Median (IQR)						9.2 (2.6–11.5)		<0.05
				Lactate	CPC 3–5 at 6 months	88	Median (IQR)						12.1 (9.5–14)		<0.05
				Lactate	CPC 1–2 at 6 months	98	AUC	Lactate < 12						0.735 (95% CI: 0.574–0.896), sensitivity 90%, specificity 52.3%	
				Lactate	CPC 1–2 at 6 months	98	OR	Lactate < 12						9.86 (95% CI 1.20–81.1)	
Sivaraju et al., 2015 [56]	2011–2014	Combined (97% OHCA)	Prospective observational cohort	pH	GOS 4–5 at discharge	29	Median (IQR)						7.31 (7.25–7.38)		<0.001
				pH	GOS 1–3 at discharge	71	Median (IQR)						7.17 (7.08–7.29)		<0.001
				Lactate	GOS 4–5 at discharge	29	Median (IQR)						3.3 (2.0–5.3)		0.003
				Lactate	GOS 1–3 at discharge	71	Median (IQR)						6.0 (3.5–10.9)		0.003
Soloperto et al., 2024 [67]	2004–2022	OHCA	Retrospective cohort	Lactate	CPC 1–2 at 3 months	160	Median (IQR)						4.5 (3.6–7.9)		<0.0001
				Lactate	CPC 3–5 at 3 months	407	Median (IQR)						7.1 (3.9–10.9)		<0.0001
Starodub et al., 2013 [57]	2005–2011	Combined (76% OHCA)	Retrospective cohort	Lactate	Survival to discharge	66	n	5	15	22.7%	51	77.3%			“NS”
				Lactate	Died prior to discharge	88	n	5	20	22.7%	68	77.3%			“NS”
				Lactate	Survival to discharge	66	n	10	42	63.6%	24	36.4%			“NS”
				Lactate	Died prior to discharge	88	n	10	52	59.1%	36	40.9%			“NS”
Takaki et al., 2013 [58]	2003–2009	OHCA	Retrospective cohort	Lactate	CPC 1–2 at 6 months	25	Mean (Range)						8.7 [3.2–17.2]		0.019
				Lactate	CPC 3–5 at 6 months	25	Mean (Range)						11.4 [5.8–21.9]		0.019
				pH	CPC 1–2 at 6 months	25	Mean (Range)						7.17 [6.894–7.4]		<0.01
				pH	CPC 3–5 at 6 months	25	Mean (Range)						6.866 [6.666–7.092]		<0.01
				pH	CPC 1–2 at 6 months	50	ROC cutoff	6.968						AUC 0.934 (95CI 0.887–0.982), sensitivity 0.92, specificity 0.84	0.01
Tetsuhara et al., 2016 [59]	2013–2015	OHCA	Retrospective case-control	pH	CPC 1–2 at discharge	13	Median (IQR)						7.22 (6.87–7.32)		0.759
				pH	CPC 3–5 at discharge	19	Median (IQR)						7.12 (6.92–7.30)		0.759
				Lactate	CPC 1–2 at discharge	13	Median (IQR)						6.6 (4.9–9.9)		0.388
				Lactate	CPC 3–5 at discharge	19	Median (IQR)						5.9 (3.5–8.9)		0.388
				pH	CPC 1–2 at discharge	13	n	7.2	6	46.2%	7	53.8%			no *p* value reported
				pH	CPC 3–5 at discharge	19	n	7.2	12	63.2%	7	36.8%			no *p* value reported
				pH	CPC 1–2 at discharge	32	OR (95% CI)							OR 0.5; 95% CI: 0.09–2.61	0.47
Tisljar et al., 2018 [60]	2012–2017	OHCA	Prospective observational cohort	Lactate	Survival to discharge	165	Median (IQR)						2.3 (1.4, 3.5)		<0.001
				Lactate	Died prior to discharge	156	Median (IQR)						4.15 (2.345, 7.3)		<0.001
				Lactate	Died prior to discharge	321	OR (95% CI)							OR (9.67; 95% CI:4.60 to 20.33)	<0.001
				Lactate	Died prior to discharge	321	AUC							0.70 95% CI: (0.65 to 0.76)	
				Lactate	CPC at discharge	321	OR (95% CI)							OR (12.58; 95% CI: 5.74 to 27.59)	<0.001
				Lactate	CPC at discharge	321	AUC							0.72 95% CI: (0.66 to 0.78)	
Tolins et al., 2017 [61]	2005–2011	Combined (75% OHCA)	Retrospective cohort	pH	CPC 1–2 at discharge	33	Mean (95% CI)						7.27 (7.22–7.31)		no *p* value reported
				pH	CPC 3–5 at discharge	81	Mean (95% CI)						7.18 (7.14–7.22)		no *p* value reported
vonAuenmueller et al., 2017 [62]	2008–2013	OHCA	Retrospective cohort	pH	Died prior to 5 days	170	OR (95% CI)	pH < 7.0						7.20 (95% CI: 3.11–16.69)	<0.001
				Lactate	Died prior to 5 days	170	OR (95% CI)	Lactate > 5						6.79 (95% CI: 2.77–16.66)	<0.001
				pH	Survival to 5 days	80	n	7	8	10.0%	72	90.0%			no *p* value reported
				pH	Died prior to 5 days	90	n	7	40	44.4%	50	55.6%			no *p* value reported
				Lactate	Survival to 5 days	79	n	5	29	36.7%	50	63.3%			no *p* value reported
				Lactate	Died prior to 5 days	89	n	5	7	7.9%	82	92.1%			no *p* value reported
Williams et al., 2016 [63]	2007–2012	OHCA	Retrospective cohort	Lactate	Survival to discharge	126	Mean (SD)						6.9 (4.7)*		<0.001
				Lactate	Died prior to discharge	392	Mean (SD)						12.2 (5.5)*		<0.001
				Lactate	Survival to discharge	126	Median (IQR)						5.9 (4.2–8.9)		<0.001
				Lactate	Died prior to discharge	392	Median (IQR)						11.1 (8.1–15.0)		<0.001
				Lactate	CPC 1–2 at discharge	518	OR (95% CI)							0.84 (95% CI 0.77–0.91)	<0.001
Yanagawa et al., 2009 [64]	2005–2007	OHCA	Retrospective cohort	pH	CPC 1–2 at one month	16	Mean (SD)						7.259 (0.036) *		<0.001
				pH	CPC 3–5 at one month	102	Mean (SD)						7.029 (0.018) *		<0.001
Zhang et al., 2021 [65]	2012–2019	OHCA	Retrospective cohort	Lactate	Survival to 28 days	476	Median (IQR)						3.50 [1.80–7.61]		<0.001
				Lactate	Died prior to 28 days	674	Median (IQR)						5.3 [2.27–10.00]		<0.001
				pH	Survival to 28 days	476	Median (IQR)						7.31 (7.17–7.40)		<0.001
				pH	Died prior to 28 days	674	Median (IQR)						7.25 (7.09–7.37)		<0.001

**Table 2:** Post-out-of-hospital-cardiac arrest lactate and pH thresholds for favorable and unfavorable survival and neurological outcomes, outliers removed. Abbreviations: CI, confidence interval; G, pooled effect size (mean); I^2^, percentage of variability in the effect size not caused by sampling error; τ^2^, variance of true effect size.

		Favorable						Unfavorable					
Exposure	Outcome	Number of Studies	N	G [95% CI]	I^2^ [95% CI]	Cochran’s Q (*p*)	τ^2^ [95% CI]	Number of Studies	N	G [95% CI]	I^2^ [95% CI]	Cochran’s Q (*p*)	τ^2^ [95% CI]
Lactate	Survival	10	1279	7.24 [6.05, 8.44]	88.4% [80.8%, 93.0%]	77.86 (<0.0001)	2.11 [0.81, 9.89]	9	2698	10.11 [8.98, 11.25]	86.1% [75.6%, 92.1%]	57.60 (<0.0001)	1.76 [0.58, 7.67]
Lactate	Neurologic Outcome	13	1005	7.15 [6.37, 7.93]	86.5% [78.6%, 91.5%]	88.96 (<0.0001)	1.31 [0.46, 4.01]	10	1738	8.76 [7.45, 10.07]	92.2% [87.7%, 95.0%]	114.69 (<0.0001)	2.88 [1.20, 11.19]
pH	Survival	6	828	7.22 [7.10, 7.33]	95.8% [93.0%, 97.4%]	117.77 (<0.0001)	0.01 [0.004, 0.07]	6	1103	7.16 [7.03, 7.29]	98.5% [97.9%, 99.0%]	335.23 (<0.0001)	0.02 [0.006, 0.09]
pH	Neurologic Outcome	9	482	7.22 [7.17, 7.27]	85.4% [74.1%, 91.7%]	54.72 (<0.001)	0.003 [0.0009, 0.013]	7	1011	7.09 [7.00, 7.18]	97.4% [96.1%, 98.2%]	230.59 (<0.0001)	0.007 [0.002, 0.05]

**Table 3:** Overview of risk of bias assessments.

Study (Author, Year)	Selection	Comparability	Outcome	Overall
Al Assil et al., 2021 [30]	Low	High	Low	Low
Carr et al., 2020 [18]	Low	Low	Low	Low
Chen et al., 2024 [73]	Low	Some concern	Low	Low
Choi et al., 2024 [72]	Low	High	Low	Low
Cocchi et al., 2020 [19]	Low	Some concern	Low	Low
Cocchi et al., 2011 [31]	Low	Some concern	Low	Low
Dell’Anna et al., 2017 [32]	Some concern	Some concern	Low	Low
Dadeh et al., 2018 [66]	Low	Some concern	Low	Low
Donnino et al., 2014 [20]	Low	Some concern	Low	Low
During et al., 2018 [33]	Low	Low	Low	Low
Dusik et al., 2023 [71]	Low	Some concern	Low	Low
FreireJorge et al., 2021 [34]	Low	Some concern	Low	Low
Han et al., 2019 [35]	Low	Some concern	Low	Low
HopeKilgannon et al., 2019 [36]	Some concern	High	Low	Medium
Imamura et al., 2023 [70]	Low	Some concern	Low	Low
Kandilcik et al, 2024 [69]	Some concern	Some concern	Low	Low
Kei et al., 2017 [37]	Low	High	Some concern	Medium
Kiehl et al., 2019 [38]	Low	High	Low	Low
Kim et al., 2023 [68]	Low	Some concern	Low	Low
Kim et al., 2017 [39]	Some concern	High	Low	Medium
Kliegel et al., 2004 [40]	Low	High	Some concern	Medium
Laurikkala et al., 2019 [41]	Low	High	Low	Low
Lee et al., 2015 [42]	Low	High	Some concern	Medium
Lin et al., 2021 [43]	Low	High	Low	Low
Lonsain et al., 2021 [44]	Low	High	Low	Low
Marinšek et al., 2020 [9]	Low	High	Low	Low
Momiyama et al., 2017 [21]	Low	High	Low	Low
Orban et al., 2017 [45]	Low	High	Low	Low
Park et al., 2019 [46]	Low	Low	Low	Low
Peluso et al., 2020 [47]	Some concern	Some concern	Low	Low
Rezar et al., 2021 [48]	Some concern	High	Low	Medium
Rosenberg et al., 2021 [49]	Some concern	Low	Low	Low
Ryoo et al., 2020 [50]	Low	High	Low	Low
Sarıaydın et al., 2017 [51]	Low	Some concern	Low	Low
Sauter et al., 2017 [52]	Low	High	Low	Low
Seeger et al., 2013 [53]	Some concern	High	Some concern	Medium
Shin et al., 2017 [54]	Low	High	Low	Low
Shinozaki et al., 2011 [55]	Low	Some concern	Low	Low
Sivaraju et al., 2015 [56]	Some concern	Some concern	Low	Low
Soloperto et al., 2024 [67]	Low	High	Low	Low
Starodub et al., 2013 [57]	Some concern	High	Low	Medium
Takaki et al., 2013 [58]	Low	Some concern	Low	Low
Tetsuhara et al., 2016 [59]	Low	Some concern	Low	Low
Tisljar et al., 2018 [60]	Low	High	Low	Low
Tolins et al., 2017 [61]	Some concern	Some concern	Low	Low
vonAuenmueller et al., 2017 [62]	Low	High	Low	Low
Williams et al., 2016 [63]	Low	High	Low	Low
Yanagawa et al., 2009 [64]	Low	Some concern	Low	Low
Zhang et al., 2021 [65]	Low	High	Low	Low

## Data Availability

The original contributions presented in this study are included in the article/Supplementary Material. Further inquiries can be directed to the corresponding author(s).

## Supplementary Materials

The following supporting information can be downloaded at https://www.mdpi.com/article/10.3390/jcm14072244/s1, Document S1: Supplemental Contents; Table S1: Detailed Collected Data for All Studies; Table S2: Detailed Risk of bias.

## Abbreviations

The following abbreviations are used in this manuscript:

AUC	Area under the curve
CPR	Cardiopulmonary resuscitation
CARES	Cardiac Arrest Registry to Enhance Survival
CPC	Cerebral Performance Category
ECPR	Extracorporeal cardiopulmonary resuscitation
EMR	Electronic medical record
EMS	Emergency medical services
GOS	Glasgow outcome scale
HR	Hazards ratio
IHCA	In-hospital cardiac arrest
IQR	Interquartile range
mRS	Modified Rankin Scale
NOS	Newcastle–Ottawa scale
OHCA	Out-of-hospital cardiac arrest
OR	Odds ratio
PICO	Population, intervention, comparison, outcome
PRISMA	Preferred Reporting Items for Systematic Reviews and Meta-Analyses
ROSC	Return of spontaneous circulation
SD	Standard deviation

## Appendix A

This appendix contains the full analysis of pH and lactate with regards to survival and neurological outcome with and without outliers, using the random effects model with Knapp–Hartung adjustment. These data are intended to be compared to the pooled analysis reported in the body of our SRMA.

### Appendix A.1. Lactate and Survival Analysis

Twenty-one articles compared lactate levels with survival outcome. Sixteen studies were retrospective cohort studies, while five were prospective observational studies for a total of 9155 patients (favorable survival, 3563; poor survival, 5592). Outcome duration ranged from hospital admission to 6-month survival. Eleven studies reported either medians or IQRs, from which means and standard deviations were deduced. In the pooled analyses for patients with lactate levels drawn after ROSC (n = 9155), a mean lactate threshold of 9.51 (95% CI: 8.36–10.66, *I*^2^ = 98.4%, Q = 1222.23 [*p* < 0.0001]) was associated with poor survival, while a mean lactate threshold of 7.16 (95% CI: 5.87–8.44; *I*^2^ = 99.1%, Q = 2184.52 [*p* < 0.0001]) was associated with favorable survival.

In the final model after outliers were removed (n = 3977), a lactate threshold of 10.11 (95% CI: 8.98–11.25, *I*^2^ = 86.1%, Q = 57.6 [*p* < 0.0001]) was associated with poor survival, while a mean lactate threshold of 7.24 (95% CI: 6.05–8.44, *I*^2^ = 88.4%, Q = 77.86 [*p* < 0.0001]) was associated with favorable survival (Table 3, Figure 2A). Detailed results are included in the Supplemental Content.

The random effects model with Knapp–Hartung Adjustment provided a pooled mean-difference effect size of 2.23 (95% CI: 1.59–2.86, *p* < 0.0001, τ^2^ = 1.41), indicating a lactate difference of nearly 2 between patients with favorable versus poor survival outcomes. After outliers were removed, the mean-difference effect size was 2.21 (95% CI: 1.76–2.65, *p* < 0.0001, τ^2^ = 0.31).

A meta-analysis of odds ratios is presented in the Supplemental Content.

### Appendix A.2. Lactate and Neurological Outcome Analysis

Twenty-one articles compared lactate levels with neurological outcome. Fifteen studies were retrospective studies, five were prospective observational studies, and one was a prospective randomized clinical trial for a total of 7534 patients (favorable neurological outcome, 1730; poor neurological outcome, 5804). Outcome duration ranged from hospital discharge to one-year neurological outcome. Fifteen studies reported either medians or IQRs, from which means and standard deviations were deduced. In the pooled analyses for patients with lactate levels drawn after ROSC (n = 7534), a mean lactate threshold of 8.82 (95% CI: 7.46–10.19, *I*^2^ = 99.7%, Q = 6819.2 [*p* < 0.0001]) was associated with poor neurological outcome, while a mean lactate threshold of 6.74 (95% CI: 5.68–7.80; *I*^2^ = 98.7%, Q = 1524.94 [*p* < 0.0001]) was associated with favorable neurological outcome.

In the final model after outliers were removed (n = 2743), a mean lactate threshold of 8.76 (95% CI: 7.45–10.07, *I*^2^ = 92.2%, Q = 114.69 [*p* < 0.0001]) was associated with poor neurological outcome, while a mean lactate threshold of 7.15 (95% CI: 6.37–7.93, *I*^2^ = 86.5%, Q = 88.96 [*p* < 0.0001]) was associated with favorable neurological outcome (Table 3, Figure 2B). Detailed results are included in the Supplemental Content.

The random effects model with Knapp–Hartung Adjustment provided a pooled mean-difference effect size of 1.99 (95% CI: 1.23–2.76, *p* < 0.0001, τ^2^ = 2.32), indicating a lactate difference of nearly 2 between patients with favorable versus poor neurological outcomes. After outliers were removed, the mean-difference effect size was 2.28 (95% CI: 1.91–2.64, *p* < 0.0001, τ^2^ = 0.19).

A meta-analysis of odds ratios is presented in the Supplemental Content.

### Appendix A.3. pH and Survival Analysis

Seven articles compared pH levels with survival outcome. Six studies were retrospective studies, while one was a prospective observational study for a total of 4077 patients (favorable survival, 1139; poor survival, 2305). Outcome duration ranged from hospital admission to 28-day survival. Four studies reported either medians or IQRs, from which means and standard deviations were deduced. In the pooled analyses for patients with pH levels drawn after ROSC (n = 4077), a mean pH threshold of 7.08 (95% CI: 6.94–7.21, *I*^2^ = 98.9%, Q = 549.55 [*p* < 0.0001]) was associated with poor survival, while a mean pH threshold of 7.18 (95% CI: 7.06–7.30; *I*^2^ = 98.3%, Q = 358.60 [*p* < 0.0001]) was associated with favorable survival.

In the final model after outliers were removed (n = 1931), a mean pH threshold of 7.16 (95% CI: 7.03–7.29, *I*^2^ = 98.5%, Q = 335.23 [*p* < 0.0001]) was associated with poor neurological outcome, while a mean pH threshold of 7.22 (95% CI: 7.10–7.33, *I*^2^ = 95.8%, Q = 117.77 [*p* < 0.0001]) was associated with favorable survival (Table 3, Figure 2C). Detailed results are included in the Supplemental Content.

The random effects model with Knapp–Hartung Adjustment provided a pooled mean-difference effect size of −0.09 (95% CI: −0.16, −0.02; *p* = 0.18; τ^2^ = 0.004), indicating a pH difference of −0.09 between patients with favorable versus poor survival outcomes. No outlier studies were identified for the random effects model.

### Appendix A.4. pH and Neurological Outcome Analysis

Thirteen articles compared pH levels with neurological outcome. Nine studies were retrospective studies, while four were prospective observational studies for a total of 6701 patients (favorable neurological outcome, 781; poor neurological outcome, 5920). Outcome duration ranged from hospital discharge to 6-month neurological outcome. Seven studies reported either medians or IQRs, from which means and standard deviations were deduced. In the pooled analyses for patients with pH levels drawn after ROSC (n = 6701), a mean pH threshold of 7.07 (95% CI: 6.99–7.14, *I*^2^ = 99.4%, Q = 1868.17 [*p* < 0.0001]) was associated with poor neurological outcome, while a mean pH threshold of 7.21 (95% CI: 7.17–7.26; *I*^2^ = 92.7%, Q = 164.84 [*p* < 0.001]) was associated with favorable neurological outcome.

In the final model after outliers were removed (n = 1493), a mean pH threshold of 7.09 (95% CI: 7.00–7.18, *I*^2^ = 97.4%, Q = 230.59 [*p* < 0.0001]) was associated with poor neurological outcome, while a mean pH threshold of 7.22 (95% CI: 7.17–7.27, *I*^2^ = 85.4%, Q = 54.72 [*p* < 0.001]) was associated with favorable neurological outcome (Table 3, Figure 2D). Detailed results are included in the Supplemental Content.

The random effects model with Knapp–Hartung Adjustment provided a pooled mean-difference effect size of −0.14 (95% CI: −0.20, −0.08; *p* = 0.0001; τ^2^ = 0.007), indicating a pH difference of nearly −0.14 between patients with favorable versus poor neurological outcomes. After outliers were removed, the mean-difference effect size was −0.13 (95% CI: −0.16, −0.10; *p* < 0.0001; τ^2^ = 0.0005).
