# Supplementary material for: Lactate and pH as Independent Biomarkers for Prognosticating Meaningful Post-out-of-Hospital Cardiac Arrest Outcomes: A Systematic Review and Meta-Analysis
Source: J Clin Med. 2025 Mar 25;14(7):2244. doi: 10.3390/jcm14072244 (PMC11989467; doi:10.3390/jcm14072244)
Supplement: Supplementary file 1 [file jcm-14-02244-s001.zip › jcm-3543340-supplementary.pdf]

SUPPLEMENTAL CONTENTS

Lactate and pH as independent biomarkers for prognosticating meaningful post-out-of-hospital cardiac arrest outcomes: a systematic review and meta-analysis

Nishil T. Patel, Casey T. Carr, Charlotte M. Hopson, Charles W. Hwang

Table of Contents

Additional supplemental content	2
Abbreviations	3
PRISMA Checklist	4
Research Protocol	7
Newcastle-Ottawa Scale for Risk of Bias	16
Meta-analyses Results	18

Additional supplemental content

The following documents are attached as separate supplemental content:

-Overview of all studies (*Excel sheet*);

-Data extraction tool (*Excel sheet*);

-Risk of bias details (*Excel sheet*);

-Appendix A;

-Data Statement.

Abbreviations

The following abbreviations are used in the supplemental content:

NOS	Newcastle–Ottawa scale
OHCA	Out-of-hospital cardiac arrest
PRISMA	Preferred Reporting Items for Systematic Reviews and Meta-Analysis
ROSC	Return of spontaneous circulation

Section and Topic	Item #	Checklist item	Location where item is reported
**TITLE**			
Title	1	Identify the report as a systematic review.	p. 1
**ABSTRACT**			
Abstract	2	See the PRISMA 2020 for Abstracts checklist.	pp. 1-2
**INTRODUCTION**			
Rationale	3	Describe the rationale for the review in the context of existing knowledge.	p. 3
Objectives	4	Provide an explicit statement of the objective(s) or question(s) the review addresses.	p. 3
**METHODS**			
Eligibility criteria	5	Specify the inclusion and exclusion criteria for the review and how studies were grouped for the syntheses.	pp. 3-4, Suppl Content
Information sources	6	Specify all databases, registers, websites, organisations, reference lists and other sources searched or consulted to identify studies. Specify the date when each source was last searched or consulted.	p. 4, Suppl Content
Search strategy	7	Present the full search strategies for all databases, registers and websites, including any filters and limits used.	Suppl Content
Selection process	8	Specify the methods used to decide whether a study met the inclusion criteria of the review, including how many reviewers screened each record and each report retrieved, whether they worked independently, and if applicable, details of automation tools used in the process.	pp. 4
Data collection process	9	Specify the methods used to collect data from reports, including how many reviewers collected data from each report, whether they worked independently, any processes for obtaining or confirming data from study investigators, and if applicable, details of automation tools used in the process.	pp. 4
Data items	10a	List and define all outcomes for which data were sought. Specify whether all results that were compatible with each outcome domain in each study were sought (e.g. for all measures, time points, analyses), and if not, the methods used to decide which results to collect.	p. 4, Suppl Content
	10b	List and define all other variables for which data were sought (e.g. participant and intervention characteristics, funding sources). Describe any assumptions made about any missing or unclear information.	p. 4, Suppl Content
Study risk of bias assessment	11	Specify the methods used to assess risk of bias in the included studies, including details of the tool(s) used, how many reviewers assessed each study and whether they worked independently, and if applicable, details of automation tools used in the process.	p. 4-5, Suppl Content
Effect measures	12	Specify for each outcome the effect measure(s) (e.g. risk ratio, mean difference) used in the synthesis or presentation of results.	pp. 5, Suppl Content
Synthesis methods	13a	Describe the processes used to decide which studies were eligible for each synthesis (e.g. tabulating the study intervention characteristics and comparing against the planned groups for each synthesis (item #5)).	pp. 5
	13b	Describe any methods required to prepare the data for presentation or synthesis, such as handling of missing summary statistics, or data conversions.	pp. 5, Suppl Content
	13c	Describe any methods used to tabulate or visually display results of individual studies and syntheses.	p. 5
	13d	Describe any methods used to synthesize results and provide a rationale for the choice(s). If meta-analysis was performed, describe the model(s), method(s) to identify the presence and extent of statistical heterogeneity, and software package(s) used.	pp. 5
	13e	Describe any methods used to explore possible causes of heterogeneity among study results (e.g. subgroup analysis, meta-regression).	pp. 5
	13f	Describe any sensitivity analyses conducted to assess robustness of the synthesized results.	pp. 5
Reporting bias assessment	14	Describe any methods used to assess risk of bias due to missing results in a synthesis (arising from reporting biases).	pp. 5

Section and Topic	Item #	Checklist item	Location where item is reported
Certainty assessment	15	Describe any methods used to assess certainty (or confidence) in the body of evidence for an outcome.	pp. 5
**RESULTS**			
Study selection	16a	Describe the results of the search and selection process, from the number of records identified in the search to the number of studies included in the review, ideally using a flow diagram.	Figure 1
	16b	Cite studies that might appear to meet the inclusion criteria, but which were excluded, and explain why they were excluded.	p. 6
Study characteristics	17	Cite each included study and present its characteristics.	Table 1, Suppl Content
Risk of bias in studies	18	Present assessments of risk of bias for each included study.	Table 2
Results of individual studies	19	For all outcomes, present, for each study: (a) summary statistics for each group (where appropriate) and (b) an effect estimate and its precision (e.g. confidence/credible interval), ideally using structured tables or plots.	Figure 2, Table 3, pp. 6-8
Results of syntheses	20a	For each synthesis, briefly summarise the characteristics and risk of bias among contributing studies.	pp. 7-8, Table 2
	20b	Present results of all statistical syntheses conducted. If meta-analysis was done, present for each the summary estimate and its precision (e.g. confidence/credible interval) and measures of statistical heterogeneity. If comparing groups, describe the direction of the effect.	pp. 6-8, Figure 2, Table 3
	20c	Present results of all investigations of possible causes of heterogeneity among study results.	pp. 7-8
	20d	Present results of all sensitivity analyses conducted to assess the robustness of the synthesized results.	pp. 6-8
Reporting biases	21	Present assessments of risk of bias due to missing results (arising from reporting biases) for each synthesis assessed.	pp. 7-8, Suppl Content
Certainty of evidence	22	Present assessments of certainty (or confidence) in the body of evidence for each outcome assessed.	pp. 6-8
**DISCUSSION**			
Discussion	23a	Provide a general interpretation of the results in the context of other evidence.	pp. 8-9
	23b	Discuss any limitations of the evidence included in the review.	pp. 9-10
	23c	Discuss any limitations of the review processes used.	pp. 9-10
	23d	Discuss implications of the results for practice, policy, and future research.	pp. 8-10
**OTHER INFORMATION**			
Registration and protocol	24a	Provide registration information for the review, including register name and registration number, or state that the review was not registered.	p. 3
	24b	Indicate where the review protocol can be accessed, or state that a protocol was not prepared.	Suppl Content
	24c	Describe and explain any amendments to information provided at registration or in the protocol.	No amendments
Support	25	Describe sources of financial or non-financial support for the review, and the role of the funders or sponsors in the review.	Suppl Content, p. 1

Section and Topic	Item #	Checklist item	Location where item is reported
Competing interests	26	Declare any competing interests of review authors.	p. 15
Availability of data, code and other materials	27	Report which of the following are publicly available and where they can be found: template data collection forms; data extracted from included studies; data used for all analyses; analytic code; any other materials used in the review.	Suppl Content

From: Page MJ, McKenzie JE, Bossuyt PM, Boutron I, Hoffmann TC, Mulrow CD, et al. The PRISMA 2020 statement: an updated guideline for reporting systematic reviews. BMJ 2021;372:n71. doi: 10.1136/bmj.n71

Lactate and pH as independent biomarkers for prognosticating meaningful post-out-of-hospital cardiac arrest outcomes: a systematic review and meta-analysis

Systematic Review Research Protocol

TITLE

Lactate and pH as independent biomarkers for prognosticating meaningful post-out-of-hospital cardiac arrest outcomes: a systematic review and meta-analysis

REGISTRATION

This protocol was registered at PROSPERO on 7 August 2024.

AUTHORS

Nishil T. Patel, D.O.

Casey T. Carr, M.D.

Charlotte M. Hopson, M.S.

Charles W. Hwang, M.D.

CORRESPONDING AUTHOR

Charles Hwang, M.D.

Department of Emergency Medicine

University of Florida College of Medicine

PO Box 100186

Gainesville, FL 32610, USA

+1 352-265-5911

hwang.c@ufl.edu

AMENDMENTS

Any major amendments or modifications to the protocol after initial registration, which may affect eligibility criteria, search strategy, data extraction, data management, outcome assessment, and data analysis and synthesis will be agreed upon by all authors and added as an amendment to the protocol. Minor administrative corrections or clarifications will not require formal documentation.

FINANCIAL SUPPORT

This systematic review was funded by the University of Florida Emergency Medicine Structured Academic Research (STAR) Grant.

INTRODUCTION

Rationale

Out-of-hospital cardiac arrest (OHCA) affects over 356,000 individuals per year in the United States [1]. Although return of spontaneous circulation (ROSC) is a crucial step in OHCA survival, ROSC is merely the first step in survival and does not necessarily confer other patient-centered outcomes such as hospital discharge or favorable neurological recovery.

Post-cardiac care is a multidisciplinary process. Contemporary research (e.g., targeted temperature management, extracorporeal cardiopulmonary resuscitation, etc.) aims to improve survival. However, it is critically important to identify appropriate patients that have a high likelihood of favorable neurological recovery. Being purposeful about which post-ROSC patients are likely to have favorable outcomes is important in alleviating the tremendous burden on the patient, healthcare systems, and families. Determining if a correlation exists between initial lactate and pH levels with favorable neurological survival can help physicians and families decide what the best options are for these patients.

Lactate is a well-known biomarker for organ perfusion status. Its importance is outlined in its robust use in the resuscitation of critically ill patients presenting with sepsis, hemorrhagic shock, cardiogenic shock, and other shock states.

We seek to determine if (and how) lactate and pH levels after ROSC is achieved can prognosticate the likelihood of meaningful neurological survival. Adding these data points to the repository of information and tools physicians use to discuss prognosis can prove useful when minutes matter. Our project aims to answer the question above by conducting a systematic review and meta-analysis of the literature.

Objectives (PICO question)

<u>Population</u>: Adult individuals with return of spontaneous circulation after nontraumatic out-of-hospital cardiac arrest.

<u>Interventions</u>: Higher serum pH and/or lower serum lactate after return of spontaneous circulation

<u>Control</u>: Lower serum pH and/or higher serum lactate after return of spontaneous circulation

Outcomes: Clinical outcomes, including, but not limited to survival/survival with a favorable or unfavorable neurological outcome at hospital discharge, and survival/survival with a favorable or unfavorable neurological outcome after hospital discharge (e.g., 30 days, 1 month, 3 months, 1 year, etc.). Final included outcomes will depend on the available data after literature search.

METHODS

Eligibility criteria

Randomized controlled trials, non-randomized controlled trials, and observational studies (prospective and retrospective, cohort and case-control studies) with a comparison group (i.e., higher lactate vs. lower lactate, higher pH vs. lower pH, favorable vs. poor survival, favorable vs. poor neurological outcome) will be included.

Animal studies, reviews, abstracts, editorials, comments, and letters to the editor will not be included. Studies using intra-arrest laboratory values to predict ROSC will not be included. Studies evaluating serum pCO2 or analyzing labs drawn prior to extracorporeal cardiopulmonary resuscitation (ECPR) initiation will not be included. Studies primarily focusing on in-hospital cardiac arrest (IHCA) (>50% IHCA population) or including pediatric patients will not be included. There will be no limitations on publication period or manuscript language (provided an English abstract existed).

The population includes adult patients with OHCA. The definition of adult patient will be dependent on the individual study, but generally will refer to individuals ≥ 18 years of age.

Information sources

We will search the following electronic databases: PubMed, Embase, Web of Science, Cochrane Central, and Academic Search Premier. The bibliographies of included articles will be reviewed for additional potential articles.

Search strategy

PubMed

(heart arrest[MeSH Terms]) OR (cardiac arrest, out of hospital[MeSH Terms]) OR (cardiopulmonary resuscitation[MeSH Terms]) OR (cardiac arrest[Title/Abstract]) OR (heart arrest[Title/Abstract]) OR ("cardiopulmonary resuscitation"[Title/Abstract])

AND

(blood gas analysis[MeSH Terms]) OR (hydrogen ion concentration[MeSH Terms]) OR (capnography[MeSH Terms)) OR (capnographies[MeSH Terms]) OR (lactates[MeSH Terms]) OR ("blood gas analysis"[Title/Abstract]) OR ("hydrogen ion concentration"[Title/Abstract]) OR (capnograph*[Title/Abstract]) OR (lactate*[Title/Abstract]) OR ("hydrogen ion concentrat*"[Title/Abstract]) OR ("end tidal CO2"[Title/Abstract) OR ("low ph"[Title/Abstract]) OR (etCO2[Title/Abstract])

<u>Embase</u>

('heart arrest'/exp OR 'out of hospital cardiac arrest'/exp OR 'resuscitation'/exp OR 'cardiac arrest')

AND

('blood gas analysis'/exp OR 'ph'/exp OR 'capnometry'/exp OR capnography OR 'lactates blood'/exp OR 'lactate blood level'/exp OR 'low ph' OR 'end tidal carbon dioxide tension'/exp)

<u>Web of Science</u>

KP=("heart arrest") OR KP=("cardiac arrest") OR KP=("Cardiopulmonary resuscitation") OR AK=("heart arrest") OR AK=("cardiac arrest") OR AK=("Cardiopulmonary resuscitation")

AND

KP=(capnography) OR KP=("capnographies) OR KP=(latate) OR KP=(lactates) OR KP=("blood gas analysis") OR KP=(hydrogen ion concentration") OR KP=("end tidal CO2") OR KP=("low ph") OR AK=(capnography) OR AK=("capnographies) OR AK=(latate) OR AK=(lactates) OR AK=("blood gas analysis") OR AK=(hydrogen ion concentration") OR AK=("end tidal CO2") OR AK=("low ph")

<u>Cochrane Central</u>

(heart arrest OR cardiac arrest OR cardiopulmonary resuscitation):ti,ab,kw

AND

("capnography" OR capnographies OR lactate OR lactates OR "blood gas analysis" OR "hydrogen ion concentration" OR "end tidal CO2" OR "low ph"):ti,ab,kw

<u>Academic Search Premier</u>

“heart arrest” OR “cardiac arrest” OR “cardiopulmonary resuscitation”

AND

"capnography" OR capnographies OR lactate OR lactates OR "blood gas analysis" OR "hydrogen ion concentration" OR "end tidal CO2" OR "low ph")

Data management

Covidence (Covidence systematic review software, Veritas Health Innovation, Melbourne, Australia. Available at www.covidence.org, 2024) will be used for article screening and full-text review. Meta-analysis of means by outcome type will be performed using the meta package in R using the restricted maximum-likelihood estimator and the inverse variance method.

Selection process

Using pre-determined screening criteria, pairs of reviewers will independently screen all titles and abstracts retrieved by the systematic review query. Any discrepancies regarding inclusion and exclusion of screened articles will be resolved by discussion between the reviewer pair, with a third reviewer adjudicating unresolved discrepancies. Kappa statistics will be calculated to determine inter-rater agreement.

Full text reports of all potentially relevant articles passing primary screening will be assessed by a pair of reviewers. Any disagreement or discrepancies will be resolved by discussion between the reviewer pair, with a third reviewer adjudicating unresolved discrepancies.

A Preferred Reporting Items for Systematic Reviews and Meta-Analysis (PRISMA) diagram showing the number of studies at each stage will be presented in the final report. This PRISMA diagram will also present reasons for exclusion of full text articles.

Data collection process

Using a predefined data extraction tool, data pertinent to the PICO will be extracted from each included article by a pair of reviewers. Missing data will be calculated from provided data if possible. Discrepancies in the extracted data will be resolved by discussion and consensus decision.

Data items

The following data will be extracted:

- General information:
  - First author name;
  - Year of publication;
  - PubMed ID;
  - Geographical location of the study (continent, country, region, city, hospital);
  - Years of patient inclusion;
  - Study design;
  - Inclusion and exclusion criteria;
  - Number of patients analyzed.
- Participants:
  - Summary demographics:
    - Age (mean, median);
    - Gender (proportion of males);
  - Location of the cardiac arrest (OHCA, proportion of OHCA);
  - Shockable/non-shockable proportion.
- Exposure and Outcomes:
  - Exposure (pH or lactate);
  - Time interval;
  - Definition of outcome;
  - Sample size of outcome;
  - Mean/median of outcome measures (pH, lactate);
  - Other relevant results (odds ratios, hazards ratios, etc.);
  - P-values.

Outcomes

The focus will be on clinical outcomes, which include but are not limited to, survival to hospital discharge, survival after hospital discharge, neurological outcome at hospital discharge, and neurological outcome after hospital discharge. The final outcomes will be dependent on the available data.

Risk of bias in individual studies

Given the nature of the PICO, it is projected that most included articles will be nonrandomized studies. The Newcastle–Ottawa scale (NOS) is an instrument developed to systematically assess quality for nonrandomized studies in a systematic review [2]. Therefore, for each included article, two authors will independently evaluate the risk of bias using the NOS, and disagreements regarding quality scoring will be resolved by discussion.

The NOS tool assesses bias in three separate domains, including (1) selection of the exposed and non-exposed cohorts, (2) comparability of the cohorts, and (3) adequacy and assessment of the outcomes.

Heterogeneity and data synthesis

Using the extracted data, the main outcome measures of interest (i.e., survival and neurological outcome) will be dichotomized into favorable versus poor (e.g., favorable survival, poor survival, favorable neurological outcome, poor neurological outcome). Means and standard deviations, if available, will be extracted and used for effect size. If the mean is available but not the standard deviation, the standard deviation will be estimated using the reported p-value or interquartile range. For studies only reporting medians and interquartile ranges, methods from Luo et al. [3] will be used to estimate the mean, and methods from Wan et al. [4] will be used to estimate the standard deviation.

Mean analysis

A meta-analysis of means (pH and lactate) by outcome type will be performed using the meta package in R by pooling the raw means and standard deviations using the restricted maximum-likelihood estimator and the inverse variance method. Random-effects models for the comparative outcome groups will be run, and the Hartung–Knapp adjustment will be applied. A “full” meta-analysis using all studies will be performed. Outlier studies will be removed from the final model using the Outliers function from the dmetar package.

A meta-analysis of standardized mean differences (pH and lactate) between outcomes will be analyzed. The effect sizes and standard errors for each study will be precalculated using the means and standard deviations; the meta-analysis will be performed using the restricted maximum-likelihood estimator and the inverse variance method in random-effects models.

Odds Ratio Analysis

Using studies that reported pH and lactate odds ratios and adjusted odds ratios for outcomes, a meta-analysis will be performed for pH and lactate odds ratios. Extracted odds ratios and confidence intervals will be log-transformed. The log-transformed odds ratios and log-transformed confidence intervals will be pooled using the inverse

variance method in random-effects models. The Paule–Mandel estimator will be used to calculate τ^2. Outlier studies will be removed using the Outliers function from the dmetar package.

REFERENCES

1. Virani SS, Alonso A, Aparicio HJ, Benjamin EJ, Bittencourt MS, Callaway CW, Carson AP, Chamberlain AM, Cheng S, Delling FN, et al. Heart Disease and Stroke Statistics-2021 Update: A Report From the American Heart Association. *Circulation*. 2021;143:e254-e743. doi: 10.1161/CIR.0000000000000950
2. *Wells G*, *Shea B*, *O'Connell D*, *Peterson J*, *Welch V*, *Losos M*, *Tugwell P*. The Newcastle-Ottawa Scale (NOS) for assessing the quality of nonrandomised studies in meta-analyses. Ottawa Hospital Research Institute. https://www.ohri.ca/programs/clinical_epidemiology/oxford.asp. 2021. Accessed July 7.
3. Luo D, Wan X, Liu J, Tong T. Optimally estimating the sample mean from the sample size, median, mid-range, and/or mid-quartile range. Stat Methods Med Res. 2018;27:1785-1805. doi: 10.1177/0962280216669183
4. Wan X, Wang W, Liu J, Tong T. Estimating the sample mean and standard deviation from the sample size, median, range and/or interquartile range. BMC Med Res Methodol. 2014;14:135. doi: 10.1186/1471-2288-14-135

NEWCASTLE - OTTAWA QUALITY ASSESSMENT SCALE
CASE CONTROL STUDIES

Note: A study can be awarded a maximum of one star for each numbered item within the Selection and Exposure categories. A maximum of two stars can be given for Comparability.

Selection

1) Is the case definition adequate?
   a) yes, with independent validation ✵
   b) yes, eg record linkage or based on self reports
   c) no description

2) Representativeness of the cases
   a) consecutive or obviously representative series of cases ✵
   b) potential for selection biases or not stated

3) Selection of Controls
   a) community controls ✵
   b) hospital controls
   c) no description

4) Definition of Controls
   a) no history of disease (endpoint) ✵
   b) no description of source

Comparability

1) Comparability of cases and controls on the basis of the design or analysis
   a) study controls for _______________ (Select the most important factor.) ✵
   b) study controls for any additional factor ✵ (This criteria could be modified to indicate specific control for a second important factor.)

Exposure

1) Ascertainment of exposure
   a) secure record (eg surgical records) ✵
   b) structured interview where blind to case/control status ✵
   c) interview not blinded to case/control status
   d) written self report or medical record only
   e) no description

2) Same method of ascertainment for cases and controls
   a) yes ✵
   b) no

3) Non-Response rate
   a) same rate for both groups ✵
   b) non respondents described
   c) rate different and no designation

NEWCASTLE - OTTAWA QUALITY ASSESSMENT SCALE
COHORT STUDIES

Note: A study can be awarded a maximum of one star for each numbered item within the Selection and Outcome categories. A maximum of two stars can be given for Comparability

Selection

1) Representativeness of the exposed cohort
   a) truly representative of the average _______________ (describe) in the community ✵
   b) somewhat representative of the average ______________ in the community ✵
   c) selected group of users eg nurses, volunteers
   d) no description of the derivation of the cohort

2) Selection of the non exposed cohort
   a) drawn from the same community as the exposed cohort ✵
   b) drawn from a different source
   c) no description of the derivation of the non exposed cohort

3) Ascertainment of exposure
   a) secure record (eg surgical records) ✵
   b) structured interview ✵
   c) written self report
   d) no description

4) Demonstration that outcome of interest was not present at start of study
   a) yes ✵
   b) no

Comparability

1) Comparability of cohorts on the basis of the design or analysis
   a) study controls for _____________ (select the most important factor) ✵
   b) study controls for any additional factor ✵ (This criteria could be modified to indicate specific control for a second important factor.)

Outcome

1) Assessment of outcome
   a) independent blind assessment ✵
   b) record linkage ✵
   c) self report
   d) no description

2) Was follow-up long enough for outcomes to occur
   a) yes (select an adequate follow up period for outcome of interest) ✵
   b) no

3) Adequacy of follow up of cohorts
   a) complete follow up - all subjects accounted for ✵
   b) subjects lost to follow up unlikely to introduce bias - small number lost - > ____ % (select an adequate %) follow up, or description provided of those lost) ✵
   c) follow up rate < ____% (select an adequate %) and no description of those lost
   d) no statement

Statistical Analyses:

Section 1: Lactate by Neurological Outcome

- Table 1: Study Summary;
- Table 1A: Meta-Analysis of Means (Poor Neurological Outcomes);
- Figure 1A: Forest Plot of Means (Poor Neurological Outcomes);
- Table 1B: Meta-Analysis of Means (Good Neurological Outcomes);
- Figure 1B: Forest Plot of Means (Good Neurological Outcomes);
- Table 1C: Meta-Analysis of Mean Differences;
- Table 1D: Meta-Analysis of Odds Ratios;
- Figure 1C: Forest Plot of Odds Ratios.
-

Section 2: Lactate by Survival Outcome

- Table 2: Study Summary;
- Table 2A: Meta-Analysis of Means (Poor Survival Outcomes);
- Figure 2A: Forest Plot of Means (Poor Survival Outcomes);
- Table 2B: Meta-Analysis of Means (Good Survival Outcomes);
- Figure 2B: Forest Plot of Means (Good Survival Outcomes);
- Table 2C: Meta-Analysis of Mean Differences;
- Table 2D: Meta-Analysis of Odds Ratios;
- Figure 2C: Forest Plot of Odds Ratios.
-

Section 3: PH by Neurological Outcome

- Table 3: Study Summary;
- Table 3A: Meta-Analysis of Means (Poor Neurological Outcomes);
- Figure 3A: Forest Plot of Means (Poor Neurological Outcomes);
- Table 3B: Meta-Analysis of Means (Good Neurological Outcomes);
- Figure 3B: Forest Plot of Means (Good Neurological Outcomes);
- Table 3C: Meta-Analysis of Mean Differences.
-

Section 4: PH by Survival Outcome

- Table 4: Study Summary;
- Table 4A: Meta-Analysis of Means (Poor Survival Outcomes);
- Figure 4A: Forest Plot of Means (Poor Survival Outcomes);
- Table 4B: Meta-Analysis of Means (Good Survival Outcomes);
- Figure 4B: Forest Plot of Means (Good Survival Outcomes);
- Table 4C: Meta-Analysis of Mean Differences.

Variable and Outcome of Interest	Mean Meta-Analysis Suggested Cutoffs (based on 95% confidence intervals for g value)	Mean Difference Model Significant?
Lactate & Poor Neurological Outcome	Sensitive Cutoff: >=7.5<br>Specific Cutoff: >=7.9	Yes
Lactate & Poor Survival Outcome	Sensitive Cutoff: >=8.4<br>Specific Cutoff: >=9.0	Yes
PH & Poor Neurological Outcome	Universal Cutoff: <=7.2	Yes
PH & Poor Survival Outcome	Sensitive Cutoff: <=7.3<br>Specific Cutoff: <=7.1	No

Section 1: Lactate by Neurological Outcome

Table 1: Study Summary:

Study ID	Author, Year	Study Type	Outcome	Outcome Time Point	Total N	Mean or SD Deduced
Mean Analysis:						
133	Dell'Anna, 2017	Prospective Observational Cohort	CPC	3 months	236	X
144	Donnino, 2014	Retrospective Cohort	mRS	Discharge	100	X
5389	Kiehl, 2019	Prospective Observational Cohort	CPC	Discharge	723	
5420	Kliegel, 2004	Retrospective Cohort	CPC	6 months	394	X
5495	Laurikkala, 2019	Prospective Observational Cohort	CPC	1 year	458	X
336	Lee, 2015	Retrospective Cohort	CPC	Discharge	443	
5688	Momiyama, 2017	Retrospective Cohort	CPC	Discharge	372	
427	Orban, 2017	Retrospective Cohort	CPC	Discharge	272	X
5839	Park, 2019	Retrospective Cohort	CPC	Discharge	102	X
441	Peluso, 2020	Retrospective Cohort	CPC	3 months	356	X
493	Ryoo, 2020	Retrospective Cohort	CPC	28 days	160	X
6097	Shin, 2017	Retrospective Cohort	CPC	28 days	2,229	

529	Shinozaki, 2011	Prospective Observational Cohort	CPC	6 months	98	X
535	Sivaraju, 2015	Prospective Observational Cohort	GOS	Discharge	100	X
6202	Takaki, 2013	Retrospective Cohort	CPC	6 months	50	X
6227	Tetsuhara, 2016	Retrospective Case-Control Analysis	CPC	Discharge	32	X
1515	Chen, 2024	Retrospective Cohort	CPC	30 days	219	
1491	Choi, 2024	Retrospective Cohort	CPC	6 months	347	X
1383	Dusik, 2023	Prospective Randomized Clinical Trial	CPC	30-180 days	82	X
1124	Imamura, 2023	Retrospective Cohort	mRS	30 days	194	
335	Soloperto, 2024	Retrospective Cohort	CPC	3 months	567	X
Total	21 studies				7,534	
Odds Ratio Analysis:						
5398	Kim, 2017	Retrospective Cohort	CPC	Discharge	282	-
441	Peluso, 2020	Retrospective Cohort	CPC	3 months	356	-
628	Williams, 2016	Retrospective Cohort	CPC	Discharge	518	-

Table 1A: Meta-Analysis of Means (Poor Neurological Outcomes):

Model	Study Number	N	G [95% CI]	Prediction Interval	I^2 [95% CI]	Cochran's Q (P)	Tau^2 [95% CI]
All Studies	21	5,804	8.8240 [7.4579, 10.1901]	2.4494, 15.1986	99.7%	6819.20 (0)	8.8442 [5.0761, 18.4994]
Outliers Removed	10	1,738	8.7628 [7.4549, 10.0708]	4.6343, 12.8913	92.2% [87.7%, 95.0%]	114.69 (<0.0001)	2.8843 [1.1996, 11.1943]
Skewed Estimates Removed	8	1,260	9.1979 [7.6887, 10.7070]	4.9137, 13.4820	89.5% [81.6%, 94.0%]	66.36 (<0.0001)	2.6864 [0.9728, 14.0159]

Figure 1A: Forest Plot of Means (Poor Neurological Outcomes, non-outlier studies):

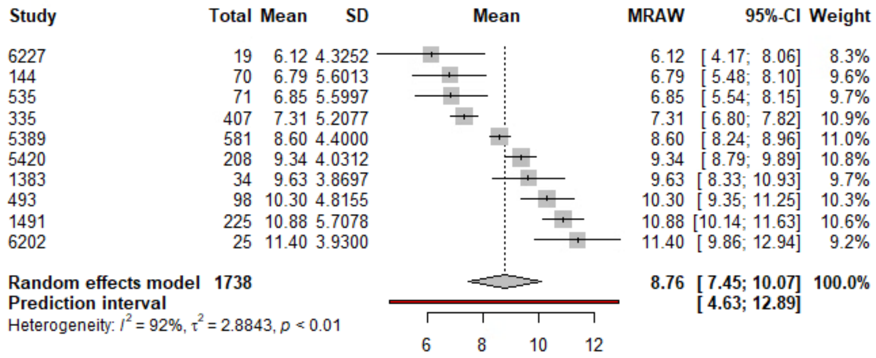

Table 1B: Meta-Analysis of Means (Good Neurological Outcomes):

Model	Study Number	N	G [95% CI]	Prediction Interval	I^2 [95% CI]	Cochran's Q (P)	Tau^2 [95% CI]
All Studies	21	1,730	6.7411 [5.6816, 7.8006]	1.8536, 11.6285	98.7% [98.4%, 98.9%]	1524.94 (<0.0001)	5.1846 [2.8192, 10.6474]
Outliers Removed	13	1,005	7.1491 [6.3662, 7.9320]	4.5025, 9.7957	86.5% [78.6%, 91.5%]	88.96 (<0.0001)	1.3086 [0.4628, 4.0061]
Skewed Estimates Removed	10	717	7.6587 [6.9658, 8.3516]	5.5882, 9.7292	77.5% [58.7%, 87.7%]	39.99 (<0.0001)	0.6912 [0.1421, 2.4079]

Figure 1B: Forest Plot of Means (Good Neurological Outcomes, non-outlier studies):

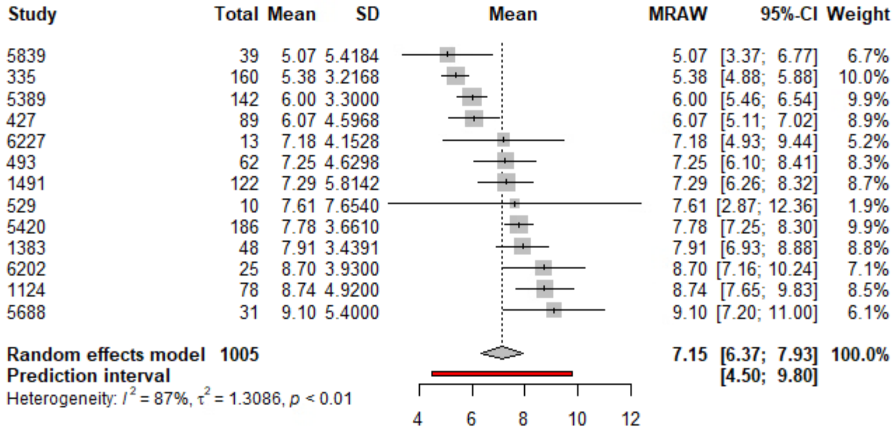

Table 1C: Meta-Analysis of Mean Differences:

Random Effects Model (with Knapp–Hartung Adjustment)			
Analysis:		All Studies	Outliers Removed
Number of Studies:		21	20
Observations (n)	Good Neurological Outcome:	1,730	1,641
	Poor Neurological Outcome:	5,804	5,621
g [95% CI]:		1.9909 [1.2262, 2.7556]	2.2764 [1.9118, 2.6411]
Model P-Value:		<0.0001	<0.0001
Prediction Interval:		[-1.2908, 5.2726]	[1.2863, 3.2666]
Tau [95% CI]		2.3043 [1.0630, 2.3043]	0.4413 [0.1813, 1.1840]
Tau^2 [95% CI]		2.3204 [1.1300, 5.3100]	0.1948 [0.0329, 1.4019]
I^2 [95% CI]		87.9% [82.8%, 91.4%]	60.6% [35.8%, 75.8%]
H [95% CI]		2.87 [2.41, 3.41]	1.59 [1.25, 2.03]
Measure of Heterogeneity (p)		Cochran's Q = 164.75 (p < 0.0001)	Cochran's Q = 48.23 (p = 0.0002)

Table 1D: Meta-Analysis of Odds Ratios:

Model	Study Number	N	Odds Ratio [95% CI]	Prediction Interval	I^2 [95% CI]	Cochran's Q (P)	Tau^2 [95% CI]
All Studies	3	1,156	1.1308 [1.0473, 1.2209]	0.5035, 2.5393	56.6% [0.0%, 87.6%)	4.61 (0.0999)	0.0025 [0.0000, 0.1737]
No Outlier Studies Identified							

Figure 1C: Forest Plot of Odds Ratios:

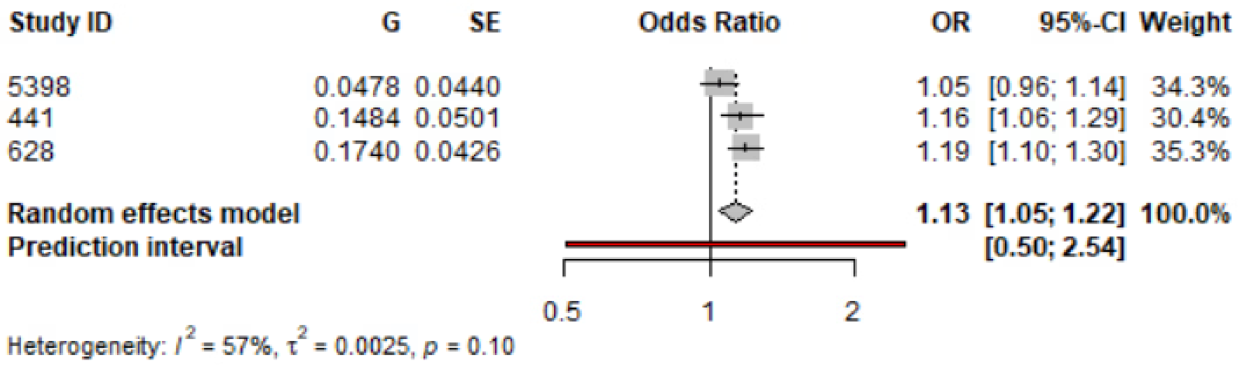

Section 2: Lactate by Survival Outcome

Table 2: Study Summary:

Study ID	Author, Year	Study Type	Outcome	Outcome Time Point	Total N	Mean or SD Deduced
Mean Analysis:						
1325	Carr, 2020	Retrospective Cohort	Survival Status	Discharge	79	
114	Cocchi, 2020	Prospective Observational Cohort	Survival Status	Discharge	249	X
144	Donnino, 2014	Retrospective Cohort	Survival Status	Discharge	100	X
153	During, 2018	Retrospective Cohort	Survival Status	30 days	877	X
182	FreireJorge	Retrospective Cohort	Survival Status	Discharge	155	X
223	Han, 2019	Prospective Observational Cohort	Survival Status	Discharge	335	
299	Kei, 2017	Prospective Observational Cohort	Survival Status	30 days	543	
5420	Kliegel, 2004	Retrospective Cohort	Survival Status	6 months	394	X
336	Lee, 2015	Retrospective Cohort	Survival Status	Discharge	443	
2000	Lonsain, 2021	Retrospective Cohort	Survival Status	72 hours	192	X
441	Peluso, 2020	Retrospective Cohort	Survival Status	Discharge	356	X
2374	Rosenberg, 2021	Retrospective Cohort	Survival Status	Discharge	50	
501	Sarıaydın, 2017	Prospective Observational Cohort	Survival Status	24 hours	140	
503	Sauter, 2017	Retrospective Cohort	Survival Status	Admission	228	
6097	Shin, 2017	Retrospective Cohort	Survival Status	Discharge	2,229	X
579	Tisljar, 2018	Prospective Observational Cohort	Survival Status	Discharge	321	X
628	Williams, 2016	Retrospective Cohort	Survival Status	Discharge	518	
6494	Zhang, 2021	Retrospective Cohort	Survival Status	28 days	1,150	X

4905	Dadeh, 2018	Retrospective Cohort	Survival Status	Discharge	207	
1048	Kandilcik, 2024	Retrospective Cohort	Survival Status	Discharge	151	X
991	Kim, 2023	Retrospective Cohort	Survival Status	Discharge	438	
Total	21 studies				9,155	
Odds Ratio Analysis:						
5398	Kim, 2017	Retrospective Cohort	Survival Status	Discharge	282	-
441	Peluso, 2020	Retrospective Cohort	Survival Status	Discharge	356	-
2374	Rosenberg, 2021	Retrospective Cohort	Survival Status	Discharge	50	-

Table 2A: Meta-Analysis of Means (Poor Survival Outcomes):

Model	Study Number	N	G [95% CI]	Prediction Interval	I^2 [95% CI]	Cochran's Q (P)	Tau^2 [95% CI]
All Studies	21	5,592	9.5058 [8.3566, 10.6551]	4.1716, 14.8400	98.4% [98.0%, 98.6%]	1222.23 (< 0.0001)	6.1865 [3.4829, 12.8899]
Outliers Removed	9	2,698	10.1129 [8.9753, 11.2505]	6.7601, 13.4657	86.1% [75.6%, 92.1%]	57.60 (< 0.0001)	1.7645 [0.5811, 7.6724]

Figure 2A: Forest Plot of Means (Poor Survival Outcomes, non-outlier studies)

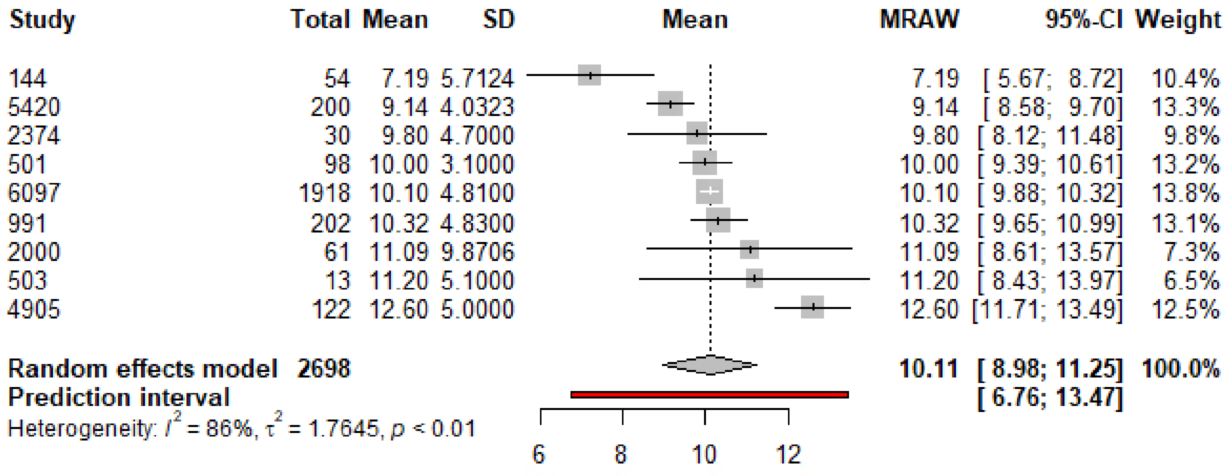

Table 2B: Meta-Analysis of Means (Good Survival Outcomes):

Model	Study Numbe r	N	G [95% CI]	Predictio n Interval	I^2 [95% CI]	Cochran' s Q (P)	Tau^2 [95% CI]
All Studies	21	3,563	7.1559 [5.8739, 8.4379]	1.2226, 13.0893	99.1% [98.9%, 99.2%]	2184.52 (0)	7.6561 [4.3443, 16.1811]
Outliers Removed	10	1,279	7.2444 [6.0465, 8.4423]	3.6972, 10.7916	88.4% [80.8%, 93.0%]	77.86 (<0.0001)	2.1101 [0.8062, 9.8900]

Figure 2B: Forest Plot of Means (Good Survival Outcomes, non-outlier studies):

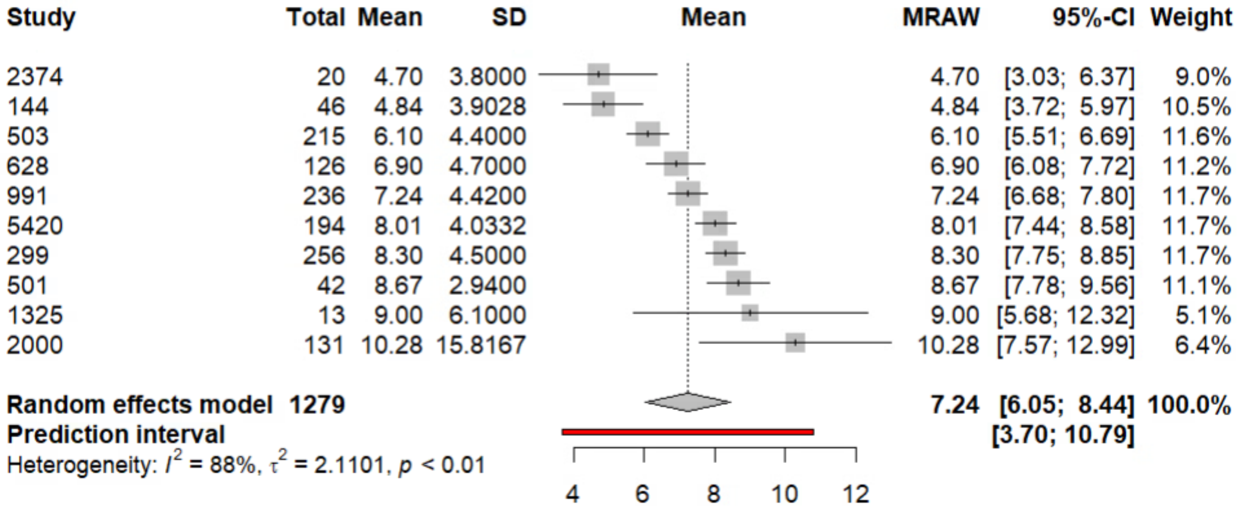

Table 2C: Meta-Analysis of Mean Differences:

**Random Effects Model (with Knapp–Hartung Adjustment)**			
Analysis:		All Studies	Outliers Removed
Number of Studies:		21	18
Observations (n)	Good Neurological Outcome:	3,563	3,044
	Poor Neurological Outcome:	5,592	3,209
g [95% CI]:		2.2262 [1.5940, 2.8584]	2.2072 [1.7625, 2.6520]
Model P-Value:		<0.0001	<0.0001
Prediction Interval:		[-0.3341, 4.7865]	[0.9517, 3.4628]
Tau [95% CI]		1.1867 [0.8251, 1.9457]	0.5611 [0.3106, 1.4788]
Tau^2 [95% CI]		1.4083 [0.6809, 3.7857]	0.3148 [0.0965, 2.1868]
I^2 [95% CI]		85.2% [78.6%, 89.8%]	58.4% [30.0%, 75.3%]
H [95% CI]		2.60 [2.16, 3.12]	1.55 [1.19, 1.01]
Measure of Heterogeneity (p)		Cochran's Q = 135.17 (p < 0.0001)	Cochran's Q = 40.89 (p = 0.0010)

Table 2D: Meta-Analysis of Odds Ratios:

Model	Study Number	N	Odds Ratio [95% CI]	Prediction Interval	I^2 [95% CI]	Cochran's Q (P)	Tau^2 [95% CI]
All Studies	3	688	1.1870 [0.9551, 1.4750]	0.0845, 16.6645	71.8% [4.3%, 91.7%]	7.08 (0.0290)	0.0309 [0.0000, 1.7302]
No Outlier Studies Identified							

Figure 2C: Forest Plot of Odds Ratios:

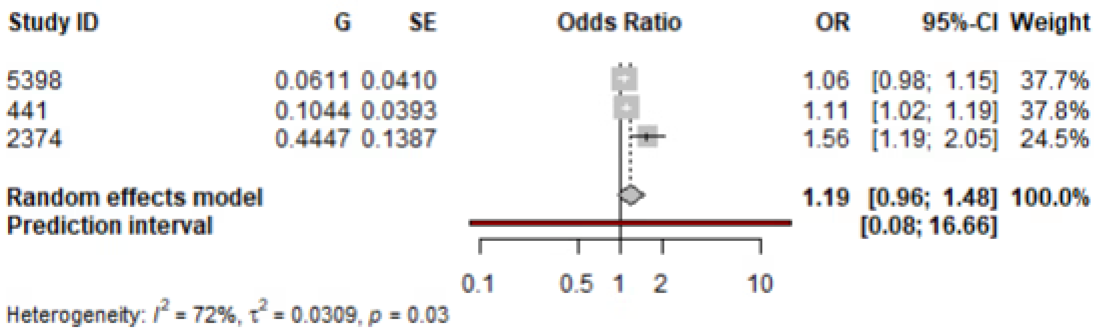

Section 3: PH by Neurological Outcome

Table 3: Study Summary

Study ID	Author, Year	Study Type	Outcome	Outcome Time Point	Total N	Mean or SD Deduced
133	Dell'Anna, 2017	Prospective Observational Cohort	CPC	3 months	236	X
5247	HopeKilgannon, 2019	Prospective Observational Cohort	mRS	Discharge	280	X
5389	Kiehl, 2019	Prospective Observational Cohort	CPC	Discharge	723	
1986	Lin, 2021	Retrospective Cohort	CPC	Discharge	2,034	
5688	Momiyama, 2017	Retrospective Cohort	CPC	Discharge	372	
6097	Shin, 2017	Retrospective Cohort	CPC	28 days	2,229	X

535	Sivaraju, 2015	Prospective Observational Cohort	GOS	Discharge	100	X
6202	Takaki, 2013	Retrospective Cohort	CPC	6 months	50	X
6227	Tetsuhara, 2016	Retrospective Case-Control Analysis	CPC	Discharge	32	X
6241	Tolins, 2017	Retrospective Cohort	CPC	Discharge	114	X
6431	Yanagawa, 2009	Retrospective Cohort	CPC	1 month	118	
1515	Chen, 2024	Retrospective Cohort	CPC	30 days	219	
1124	Imamura, 2023	Retrospective Cohort	mRS	30 days	194	
Total	13 articles				6,701	

Table 3A: Meta-Analysis of Means (Poor Neurological Outcomes):

Model	Study Number	N	G [95% CI]	Prediction Interval	I^2 [95% CI]	Cochran's Q (P)	Tau^2 [95% CI]
All Studies	13	5,920	7.0688 [6.9937, 7.1439]	6.7931, 7.3445	99.4% [99.2%, 99.5%]	1868.17 (0)	0.0145 [0.0072, 0.0418]
Outliers Removed	7	1,011	7.0885 [6.9996, 7.1773]	6.8620, 7.3149	97.4% [96.1%, 98.2%]	230.59 (<0.0001)	0.0066 [0.0022, 0.0535]

Figure 3A: Forest Plot of Means (Poor Neurological Outcomes, non-outlier studies)

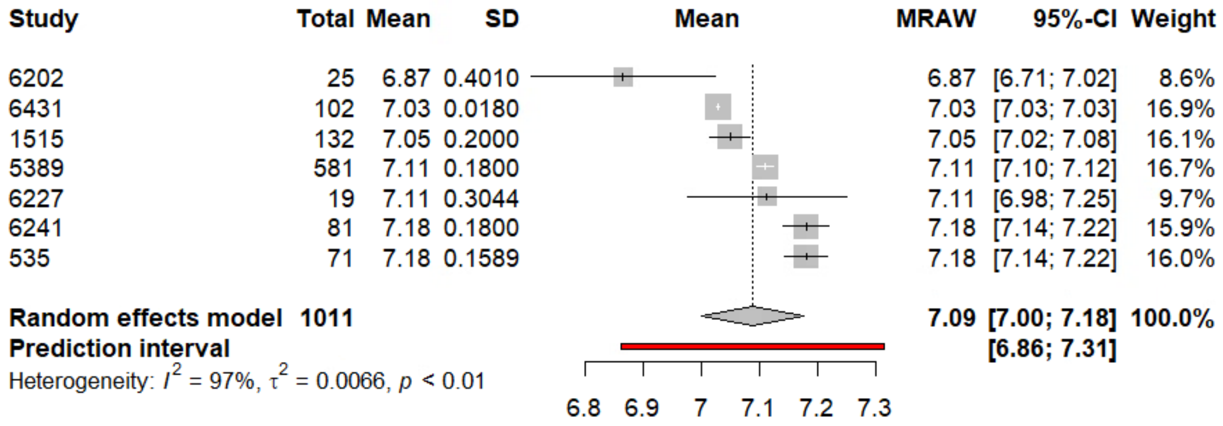

Table 3B: Meta-Analysis of Means (Good Neurological Outcomes):

Model	Study Number	N	G [95% CI]	Prediction Interval	I^2 [95% CI]	Cochran's Q (P)	Tau^2 [95% CI]
All Studies	13	781	7.2147 [7.1699, 7.2594]	7.0531, 7.3762	92.7% [89.3%, 95.0%]	164.84 (<0.001)	0.0049 [0.0021, 0.0137]
Outliers Removed	9	482	7.2186 [7.1714, 7.2657]	7.0788, 7.3584	85.4% [74.1%, 91.7%]	54.72 (<0.001)	0.0030 [0.0009, 0.0128]

Figure 3B: Forest Plot of Means (Good Neurological Outcomes, non-outlier studies)

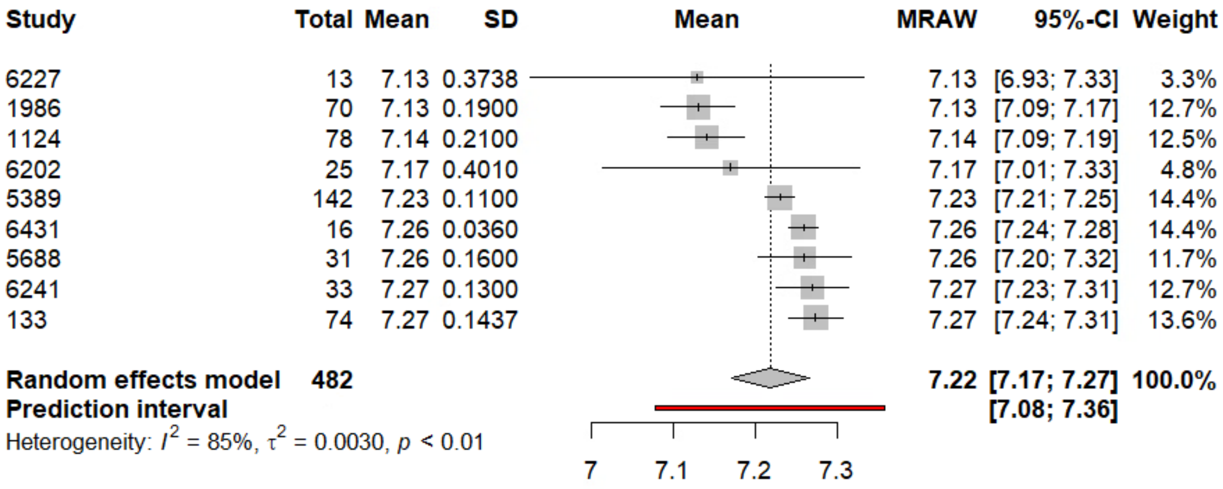

Table 3C: Meta-Analysis of Mean Differences

Random Effects Model (with Knapp–Hartung Adjustment)			
Analysis:		All Studies	Outliers Removed
Number of Studies:		13	9
Observations (n)	Good Neurological Outcome:	781	575
	Poor Neurological Outcome:	5,920	5,120
g [95% CI]:		-0.1405 [-0.1961, -0.0848]	-0.1314 [-0.1627, -0.1001]
Model P-Value:		0.0001	<0.0001
Prediction Interval:		[-0.3334, 0.0525]	[-0.1935, -0.0694]
Tau [95% CI]		0.0839 [0.0561, 0.1522]	0.0232 [0.0000, 0.1164]
Tau^2 [95% CI]		0.0070 [0.0031, 0.0232]	0.0005 [0.0000, 0.0135]
I^2 [95% CI]		94.9% [92.9%, 96.4%]	48.6% [0.0%, 76.1%]

H [95% CI]	4.44 [3.74, 5.28]	1.39 [1.00, 2.04]
Measure of Heterogeneity (p)	Cochran's Q = 236.88 (p < 0.0001)	Cochran's Q = 15.56 (p = 0.0491)

Section 4: PH by Survival Outcome

Table 4: Study Summary

Study ID	Author, Year	Study Type	Outcome	Outcome Time Point	Total N	Mean or SD Deduced
1325	Carr, 2020	Retrospective Cohort	Survival Status	Discharge	79	X
144	Donnino, 2014	Retrospective Cohort	Survival Status	Discharge	100	X
501	Sarıaydın, 2017	Prospective Observational Cohort	Survival Status	24 hours	140	
503	Sauter, 2017	Retrospective Cohort	Survival Status	Admission	228	
6097	Shin, 2017	Retrospective Cohort	Survival Status	Discharge	2,229	X
6494	Zhang, 2021	Retrospective Cohort	Survival Status	28 days	1,150	X
1048	Kandilcik, 2024	Retrospective Cohort	Survival Status	Discharge	151	
All studies	7 articles				4,077	

Table 4A: Meta-Analysis of Means (Poor Survival Outcomes):

Model	Study Number	N	G [95% CI]	Prediction Interval	I^2 [95% CI]	Cochran's Q (P)	Tau^2 [95% CI]
All Studies	7	2,305	7.0767 [6.9391, 7.2142]	[6.6714, 7.4820]	98.9% [98.5%, 99.2%]	549.55 (<0.0001)	0.0217 [0.0087, 0.1065]
Outliers Removed	6	1,103	7.1596 [7.0293, 7.2899]	[6.7902, 7.5290]	98.5% [97.9%, 99.0%]	335.23 (<0.0001)	0.0151 [0.0055, 0.0904]

Figure 4A: Forest Plot of Means (Poor Survival Outcomes, non-outlier studies)

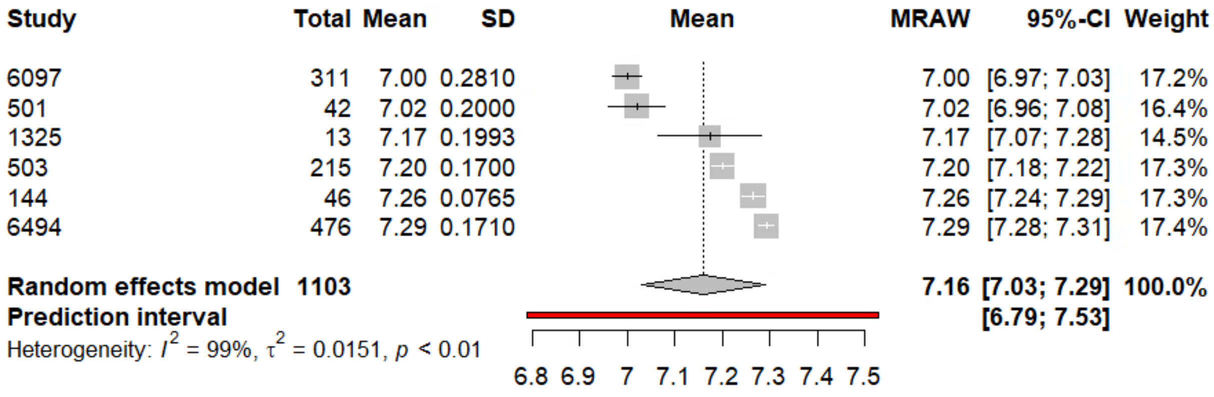

Table 4B: Meta-Analysis of Means (Good Survival Outcomes):

Model	Study Number	N	G [95% CI]	Prediction Interval	I^2 [95% CI]	Cochran's Q (P)	Tau^2 [95% CI]
All Studies	7	1,139	7.1829 [7.0639, 7.3018]	[6.8330, 7.5327]	98.3% [97.6%, 98.8%]	358.60 (<0.0001)	0.0161 [0.0064, 0.0786]
Outliers Removed	6	828	7.2152 [7.1010, 7.3294]	[6.8975, 7.5328]	95.8% [93.0%, 97.4%]	117.77 (<0.0001)	0.0111 [0.0039, 0.0707]

Figure 4B: Forest Plot of Means (Good Survival Outcomes, non-outlier studies)

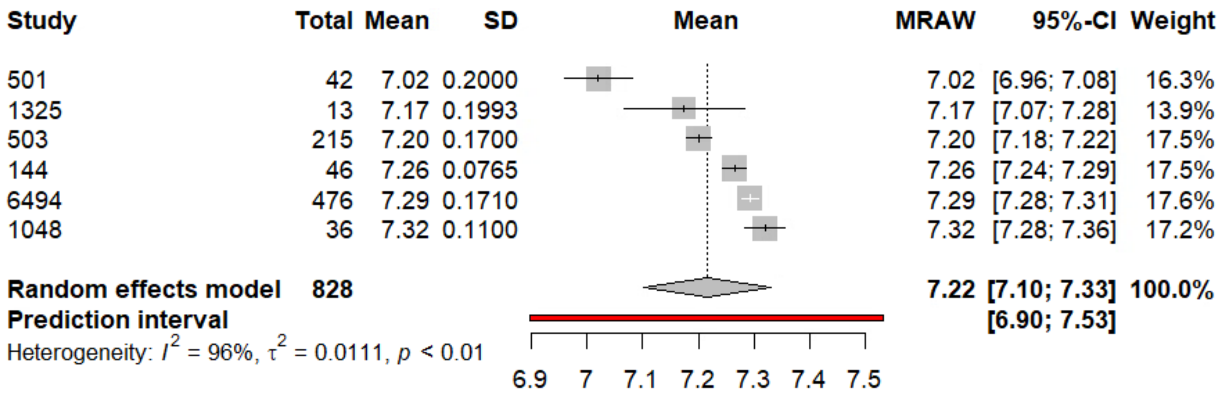

Table 4C: Meta-Analysis of Mean Differences

<table>
<tr><th colspan="4">Random Effects Model (with Knapp–Hartung Adjustment)</th></tr>
<tr><td colspan="2">Analysis:</td><td>All Studies</td><td rowspan="12">No Outlier Studies Identified</td></tr>
<tr><td colspan="2">Number of Studies:</td><td>7</td></tr>
<tr><td rowspan="2">Observations (n)</td><td>Good Neurological Outcome:</td><td>1,139</td></tr>
<tr><td>Poor Neurological Outcome:</td><td>2,305</td></tr>
<tr><td colspan="2">g [95% CI]:</td><td>-0.0936
[-0.1647, -0.0225]</td></tr>
<tr><td colspan="2">Model P-Value:</td><td>0.1808</td></tr>
<tr><td colspan="2">Prediction Interval:</td><td>[-0.2685, 0.0812]</td></tr>
<tr><td colspan="2">Tau [95% CI]</td><td>0.0626
[0.0317, 0.1808]</td></tr>
<tr><td colspan="2">Tau^2 [95% CI]</td><td>0.0039
[0.0010, 0.0327]</td></tr>
<tr><td colspan="2">I^2 [95% CI]</td><td>75.1%
[47.3%, 88.3%]</td></tr>
<tr><td colspan="2">H [95% CI]</td><td>2.00 [1.38, 2.92]</td></tr>
<tr><td colspan="2">Measure of Heterogeneity (p)</td><td>Cochran’s Q = 24.12
(p = 0.0005)</td></tr>
</table>
